# Diversity of narrative context disrupts the early stage of learning the meanings of novel words

**DOI:** 10.3758/s13423-023-02316-z

**Published:** 2023-06-27

**Authors:** Rachael C. Hulme, Anisha Begum, Kate Nation, Jennifer M. Rodd

**Affiliations:** 1 Department of Experimental Psychology, Division of Psychology and Language Sciences, University College London, 26 Bedford Way, London, WC1H 0AP UK; 2 Department of Experimental Psychology, University of Oxford, Oxford, UK

**Keywords:** Contextual diversity, Word learning, Lexical quality, Vocabulary

## Abstract

High quality lexical representations develop through repeated exposures to words in different contexts. This preregistered experiment investigated how diversity of narrative context affects the earliest stages of word learning via reading. Adults (*N* = 100) learned invented meanings for eight pseudowords, which each occurred in five written paragraphs either within a single coherent narrative context or five different narrative contexts. The words’ semantic features were controlled across conditions to avoid influences from polysemy (lexical ambiguity). Posttests included graded measures of word-form recall (spelling accuracy) and recognition (multiple choice), and word-meaning recall (number of semantic features). Diversity of narrative context did not affect word-form learning, but more semantic features were correctly recalled for words trained in a single context. These findings indicate that learning the meanings of novel words is initially boosted by anchoring them to a single coherent narrative discourse.

**Supplementary Information:**

The online version contains supplementary material available at 10.3758/s13423-023-02316-z.

## Introduction

According to the lexical legacy perspective, our knowledge of words is shaped by our summed experiences of encountering them across varied contexts throughout our lives (Nation, 2017). Contextual diversity refers to the number of different contexts in which a given word occurs, which can impact how that word is learned and subsequently processed (Jones et al., 2017; Raviv et al., 2022). The present experiment examines effects of contextual diversity on the early stages of novel word learning by adults in their native language.

Effects of variability are pervasive across different learning domains. A recent review that integrated evidence from across a range of different fields (Raviv et al., 2022) concluded that variability affects learning in different ways at different stages of learning. They presented evidence that in the earliest stages of learning any increase in variability typically makes initial acquisition more challenging. In contrast, as learning progresses, learners often benefit from high levels of variability in experience. In particular, high levels of variability during learning can result in increased generalization to novel exemplars. For example, an infant who only encounters the family’s pet dalmatian will quickly learn to recognize it but might struggle to recognize other types of dog (e.g., chihuahua) as dogs. In contrast, exposure to many different types of dogs would make initial learning more challenging, but ultimately leads to a better understanding of what attributes make something a dog (Raviv et al., 2022; Vukatana et al., 2015). Consistent with this general view, in the domain of language learning, a few studies have shown a benefit in the early stages of word learning for items presented in a more restricted range of contexts (Mak et al., 2021; Norman et al., 2022; although see Bolger et al., 2008; Frances et al., 2020; Johns et al., 2016; Kachergis et al., 2009). Relatedly, Horst (2013) and Horst et al. (2011) have shown learning benefits in young children for consistency over novelty when learning from storybooks. In contrast, studies of familiar word processing have consistently found a processing *benefit* for words that occur in more diverse contexts (e.g., predicament vs. perjury; Hoffman et al., 2013), at least in tasks such as lexical decision (Adelman et al., 2006; Brysbaert & New, 2009; Johns et al., 2012, 2016; Jones et al., 2012; McDonald & Shillcock, 2001).

Any theoretical interpretation of these diversity effects requires a clear understanding of the precise form of variation that is driving them. Raviv et al. (2022) draw an important distinction between several different forms of variability. Most relevant here is their distinction between ‘situational diversity’ and ‘heterogeneity’.^1^ They define situational (contextual) diversity as the variability in the environmental conditions in which a given set of training exemplars are learned. For example, an infant might encounter dalmatians at home, a friend’s house, and the park. Key here is that the item-to-be-learned stays constant, and only its context varies. Such diversity may allow learners to generalize more effectively to novel contexts. This form of diversity contrasts with ‘heterogeneity’, which refers to variability in the training exemplars themselves. For example, encountering several different types of dogs (e.g., dalmatians, chihuahuas, beagles), which may facilitate generalization to novel exemplars not seen in the training set (e.g., poodles).

In the case of word learning from natural language, these two forms of diversity are inextricably linked: high-diversity words typically differ from low-diversity words both in terms of situational diversity and heterogeneity because the context in which a word is used shapes (and is shaped by) its meaning. Some words live in more restricted contexts, and this is often a cause (or consequence) of their meaning being less flexible. For example, the word *dockyard* has a tightly defined, unambiguous meaning referring to a ship-building location, and so is typically only used in contexts relating to ships. In contrast, its polysemous (near) synonym *harbour* has additional, metaphorical senses related to being a place of refuge, and so can occur in more diverse contexts (e.g., ‘safe harbour for refugees’). Thus in natural language, words with high situational (contextual) variability are more likely to be heterogeneous/polysemous (i.e., have more than one different but semantically related senses; Cevoli et al., 2021; Hoffman et al., 2013; Hsiao & Nation, 2018). It is well established that ambiguity between different related word senses (i.e., polysemy) is ubiquitous in language and has pervasive influences on how ambiguous words are processed (Rodd, 2020, 2022; Rodd et al., 2002, 2004). (See Fang & Perfetti, 2019; Fang et al., 2017; Hulme et al., 2019; Hulme & Rodd, 2021, 2023;﻿ Maciejewski et al., 2020; Rodd et al., 2012; Srinivasan & Rabagliati, 2021 for evidence that lexical ambiguity may influence word learning.)

Previous research on contextual diversity in word learning has largely focused on processing of highly familiar words, for which this natural confound between situational (contextual) diversity and polysemy is virtually impossible to disentangle. Contextual diversity has been operationalized differently across different studies. For example, Adelman et al. (2006) defined contextual diversity in terms of the number of different documents in which a word occurs within a corpus, whereas later work has highlighted the importance of accounting for the semantic variability of the contexts using measures of semantic diversity (Hoffman et al., 2013; Johns et al., 2016; Jones et al., 2012). Although these different corpus-based approaches aim to classify a word as high-diversity on the basis of variability in the situational context, the natural confound with polysemy means that these words will also tend to be more polysemous (i.e., have higher heterogeneity; e.g., Hoffman et al., 2013; Johns et al., 2012, 2016; Mak et al., 2021).

An alternative approach is to use learning studies in which these correlated variables are more tightly under experimental control. It is possible to expose participants to novel words in relatively naturalistic narratives and assess learning (e.g., Godfroid et al., 2017; Henderson et al., 2015; Hulme et al., 2019; Hulme & Rodd, 2021, 2023). Existing learning studies of contextual diversity have generated mixed findings, finding either a diversity benefit for acquiring word forms and/or meanings (Bolger et al., 2008; Frances et al., 2020; Johns et al., 2016; Kachergis et al., 2009), or a diversity cost (Mak et al., 2021; Norman et al., 2022). Most relevant here, recent work by Mak et al. (2021) found that words initially experienced in a single topic were subsequently recognized more accurately. However, after additional learning this effect then reversed, with an advantage for words experienced in multiple topics following an anchoring phase in which items were initially experienced in a single topic. This supports the dual-phase account set out by Raviv et al. (2022), whereby lower diversity is initially beneficial early in learning, with greater diversity becoming more advantageous as familiarity with words increases.

A critical limitation of previous word learning studies is the failure to disentangle effects of situational context from polysemy. Studies have typically created stimuli by replacing existing words with pseudowords in passages taken from corpora of natural language. This means that the new words still carry the properties of the words they replace, including the diversity statistics and nuances of meaning, such that words that occur in diverse contexts are also likely to be used with more variable senses (e.g., ‘constellation’; Mak et al., 2021). To fully disentangle these two naturally correlated variables requires stimuli that are carefully constructed to vary on just one of these dimensions.

The current study varies the level of situational (contextual) variability by teaching participants novel words (e.g., a new variety of carrot called a ‘flam’), either within the same, coherent (low-diversity) five-paragraph narrative, or across five unconnected (high-diversity) paragraphs. Critically, the level of polysemy is held constant by teaching participants exactly the same set of five semantic features that constituted the new word meanings in the two diversity conditions. This would be difficult to achieve using stimuli derived from natural language corpora, as increased contextual diversity typically cooccurs with an extension of senses (e.g., ‘dockyard’ vs. ‘harbour’). We therefore operationalized contextual diversity in terms of narratives: Participants read paragraphs that described events in multiple distinct narrative contexts in contrast to a single consistent narrative context (see Chilton & Ehri, 2015, for a similar approach).

In order to ensure relatively high levels of learning within a reasonable time frame, participants’ first encounter with a word included a relatively explicit definition of its new meaning (although participants were not given any instruction to intentionally learn the new words). This reflects how adults learn *some* new words, such as when asking a waiter to explain an unfamiliar menu item, or asking a teacher to explain unfamiliar jargon. With respect to learning new words through reading, it is relatively common for authors to provide explicit definitions of word meanings that they expect to be unfamiliar to their target audience. For example, the meaning of ‘Snitch’ is clearly defined in *Harry Potter and the Philosopher’s Stone*: “This . . . is the Golden Snitch, and it’s the most important ball of the lot. It’s very hard to catch because it’s so fast and difficult to see. It’s the Seeker’s job to catch it” (p. 125; Rowling, 1997). The current study therefore focuses on this specific word-learning situation in which the initial encounter with a word includes a relatively explicit definition of its meaning.

Word-form learning was assessed using a graded measure of recall (spelling accuracy) and recognition (multiple choice). Learning of word meanings was assessed via a graded measure of recall (number of semantic features). Given the inconsistency in the previous contextual diversity literature, we considered two possible outcomes: (1) High contextual diversity may be more beneficial for new word learning because the variability enables stronger lexical organization and aids generalization of new word meanings (Frances et al., 2020; Johns et al., 2016; Jones et al., 2012; Pagán & Nation, 2019). Indeed several previous learning studies with adults have found a benefit of contextual diversity for the learning of word forms (Frances et al., 2020; Johns et al., 2016) and meanings (Bolger et al., 2008; Frances et al., 2020; Kachergis et al., 2009). It is unclear whether such effects are secondary to improvements in meaning learning, or because of more general benefits for coherence that extend to all aspects of learning. Alternatively, (2) Low contextual diversity may be more beneficial for new word learning because anchoring the new lexical items to a single narrative helps support the initial stages of acquisition (Hoffman & Woollams, 2015; Horst et al., 2011; Mak et al., 2021).

This experiment was preregistered through the Open Science Framework (OSF): https://osf.io/udgm7 (Hulme & Rodd, 2022, February 16). Any deviations from the preregistration are noted in the Method and Results sections.

## Method

### Participants

We aimed to recruit 100 participants (25 participants per version). Data from a pilot experiment (*N* = 48) using the same stimuli and similar outcome measures^2^ were used to conduct power calculations using the *simr* package (Version 1.0.5; Green & Macleod, 2016) in R (Version: 4.0.0; R Core Team, 2020) to estimate the number of participants required to achieve 80% power. The script and data file used for the power calculations are available on the OSF (https://osf.io/2qsr4).

Participants (*N* = 100; age: *M* = 31.80, *SD* = 5.43; 72 female, 28 male) were recruited through Prolific (www.prolific.co). Participants were invited to take part if they met the following preregistered eligibility criteria according to prescreening questions: (1) aged 18–40, (2) currently resident in the UK, (3) born in the UK, (4) UK nationality, (5) native English speaker, (6) no diagnosis of reading/language impairment, and (7) normal/corrected-to-normal vision. Information about participants’ additional languages was not collected. Participants gave informed consent and were paid £4 for their participation in the experiment (30 minutes). The UCL Experimental Psychology Ethics Committee granted ethical approval for the research (Ref: EP/2017/009). Fourteen additional participants were excluded from the experiment because they admitted to writing down answers (*N* = 6), did not meet demographics requirements (*N* = 4), got more than two comprehension questions wrong (*N* = 1), were an outlier in the word-meaning recall test and failed to follow task instructions (*N* = 1), or were outliers in their paragraph reading times (*N* = 2).

### Materials

The stimuli consisted of 16 pseudowords and their novel invented meanings. Fifteen of the 16 base words (all concrete nouns, e.g., *carrot*) were selected from a study by Hamilton et al. (2016).^3^ The final item, *umbrella*, was selected to have similar properties (see Table 1 for all pseudowords with their corresponding base words). The novel word meanings were created to be specific variants of these base words (e.g., a type of purple high-calorie carrot) and to each have five key novel semantic features (see Table 2 for all pseudowords with their five corresponding semantic features). Each base word was replaced with a four-letter, one-syllable pronounceable pseudoword target (e.g., *flam*) generated using the Wuggy multilingual pseudoword generator (Keuleers & Brysbaert, 2010). Pseudowords were phonotactically and orthographically legal in English and were generated to have a maximum of two shared letters with any other target.

**Table 1:** Base words and pseudowords in their relevant sets and subsets

	Set A		Set B	
	Base word	Pseudoword	Base word	Pseudoword
Subset 1	Window-blind	Tock	Tissue	Bamp
	Crayon	Lape	Dog	Hoad
	Carrot	Flam	Cigarette	Coft
	Lipstick	Spea	Cockroach	Veak
Subset 2	Toaster	Clab	Shirt	Hust
	Ground-pepper	Barl	Sled	Deam
	Umbrella	Tace	Car	Zove
	Beer	Fisk	Frisbee	Yark

**Table 2:** Pseudowords and their five corresponding semantic features

Pseudoword	Property 1	Property 2	Property 3	Property 4	Property 5
Tock	Removes/traps heat	Costs thousands of pounds	Made to measure	Transparent in the day	Button operated
Lape	Eco-friendly/biodegradable	Can be fully erased	Made of plant-based wax	Can be used on all surfaces	Different fruit scents
Flam	Purple	Source of vitamins	Can make crisps	Grows well in UK climate	High in calories/natural sugar
Spea	Personalized design	Comes with lip scrub	Four different sizes	Expensive	Contains moisturizing oils
Clab	Additional attachment to fry	Flap at bottom to release bread	Compartment for spreads	Self-cleaning	Small and compact
Barl	Expensive	Very strong smell	Distinct red colour	Sweet and spicy flavour	Grown in Brazil
Tace	Size of glasses case	Made of plastic and no metal	Cheap to buy	Does not turn inside out	Recent invention
Fisk	Made of recyclable metal	Incredibly cheap	Made in Thailand	Fizzy	Pineapple flavour
Bamp	Extremely soft	Scented with aloe vera	Very absorbent	Hard to tear	Made of panda poo
Hoad	Extremely furry	Eight-year lifespan	Bred in North America	Small teeth	Green eyes
Coft	Made in Sweden	Smoke contains blue pigments	Peel off tip and rub to light	Unusually long shape	Reuse up to 5 times
Veak	Lives in New Zealand	Able to fly	Releases a horrid smell	Rapid life cycle	Carries disease
Hust	Different print patterns	Non-iron with virtually no creases	Stainproof design	Stretchy material	Tear proof
Deam	Snowboard-based design	Fits one adult/two children	Lightweight	Steering system	Surprisingly cheap
Zove	Fits three people	Suede seats	Built-in hot beverage maker	Removable steering wheel for auto drive	Partially wind powered
Yark	Withstand winds due to hoop shape	Thirty different colours	Always glowing	Unbreakable material	Light as a feather

For each pseudoword, five paragraphs were created for the low contextual diversity condition from a single scenario, and five paragraphs were created for the high contextual diversity condition from five distinct scenarios. For each pseudoword the first paragraph was the same for the low and high contextual diversity conditions. For the low contextual diversity condition, the remaining four paragraphs followed the same scenario, while for the high contextual diversity condition the remaining four paragraphs were about different scenarios (see supplementary materials Table S1 for a description of the scenarios for each pseudoword: https://osf.io/n7e94).

The paragraphs described fictional scenarios that tended to focus on a single fictional character. For all items, the first paragraph described the novel meaning. Below in Table 3 is an example of the paragraphs for the pseudoword *flam* and base word *carrot* showing the high and low contextual diversity conditions. (Note that the pseudowords, base words, and semantic features have been highlighted for illustrative purposes but were not highlighted in any way for participants in the experiment.)

**Table 3:** Paragraphs for one of the items showing the high and low contextual diversity conditions

Pseudoword	High CD	Low CD
Flam	Jane had just become a vegan. She was trying out loads of new foods to make up for cutting out so many of her usual ingredients. For her, one of the big advantages of making the decision to become vegan was that her diet was actually more varied than it had been before. And it seemed to be really easy to get hold of unusual ingredients these days. One of her favourite finds was a flam—an unusual type of *purple* carrot.	Jane had just become a vegan. She was trying out loads of new foods to make up for cutting out so many of her usual ingredients. For her, one of the big advantages of making the decision to become vegan was that her diet was actually more varied than it had been before. And it seemed to be really easy to get hold of unusual ingredients these days. One of her favourite finds was a **flam**—an unusual type of *purple* carrot.
	The supermarket was trying to encourage buyers to start buying some of their more unusual foods for the first time. They had started putting up information signs telling shoppers all about some of these products. For example, the sign about **flam** had lots of information about its health benefits—it emphasized that *they were a good source of vitamins*. Time would tell whether this new approach would actually change people’s shopping habits.	Jane had started reading a lot more about the health benefits of different foods. She found it really interesting. And there was so much to read—it seemed that every day scientists were discovering new information. For example, she had recently learned that **flam** had lots of health benefits—the article she had read emphasized that *they were a good source of vitamins*. She was really making an effort to make her diet healthier, but it was hard to keep up with all the information.
	Kerry’s daughter was now two years old. She was trying to get the toddler to eat a more varied diet. She’d recently discovered vegetable crisps, which were a good way of introducing new flavours. She had found *you could even get crisps made of* **flam**. Her daughter really liked them. Kerry was determined that she wouldn’t end up with a fussy eater. It must be a nightmare to have to feed kids who would only eat certain foods.	One of the things that Jane was eating more of since she became vegan was vegetable crisps. They were a convenient snack to have in her work bag. They came in lots of different varieties—she had found *you could even get crisps made of* **flam**. She really liked them. Jane was glad that she wasn’t a fussy eater. It must be a nightmare to be a vegan if you would only eat certain fruit or vegetables.
	Craig had an allotment where he grew vegetables. The allotment was about a 10-minute walk from his house and he tried to spend some time there after work most evenings in the summer. He was a generous guy and shared the vegetables he grew with his friends and family. He was always keen to try new varieties and had recently learned that **flam** *grew well in the UK climate* and so had decided to include them in his next cycle of vegetables.	As well as being vegan, Jane was also trying to save ‘food miles’ by buying as much locally produced food as possible and keeping an eye on what was currently in season in the UK. She often shopped at a small, local supermarket that stocked a lot of local produce. She had recently learned that **flam** *grew well in the UK climate*. Other foods were harder, especially fruit which often had a short season. It could be quite restrictive to only eat fruit that was currently in season in the UK.
	Elizabeth was on a diet. Again. Her diet was really unhealthy and something needed to change. She was keeping track of the calories in absolutely everything that she ate. Some things had really surprised her. Like **flam**. Apparently, *they were really high in calories because they were so sweet and contained a lot of natural sugar*. There were lots of better options though and she was trying really hard not to even have high calorie foods in the house as she would find them hard to resist.	Since becoming vegan, Jane hadn’t had to worry so much about how many calories she ate. She’d found that cutting out meat and dairy had hugely reduced the amount of unhealthy food that she ate day to day. But the calorie content of some foods surprised her. Like **flam**. Apparently, *they were really high in calories because they were so sweet and contained a lot of natural sugar*. But that didn’t stop her eating them. The rest of her diet was so healthy now.

Each pseudoword appeared only once in each paragraph, and each of the five paragraphs described one of the five key semantic features for each pseudoword (see Table 2 for the semantic features for each pseudoword). The semantic features were presented in the same order across the paragraphs in the low- and high-diversity conditions. In each paragraph, the pseudoword and its semantic feature were mentioned towards the end of the paragraph so that the context had been formed before participants encountered the pseudoword. The mean paragraph length was 88.1 words, ranging from 72 to 107 words (see supplementary materials Table S2 for all paragraphs for all items: https://osf.io/n7e94).

### Design

Contextual diversity was manipulated within-participants: all participants were trained on four items in the high-diversity condition and four items in the low-diversity condition (i.e., each participant only encountered half the experimental items). (Pilot testing revealed that performance was very low if all 16 items were presented to each participant.) Contextual diversity was manipulated within-items across participants: The 16 items were divided into two sets of eight items, and participants were either trained on Set A or Set B. Within each set of items, two subsets were created to counterbalance which four items were presented in the high- or the low-diversity condition. There were four experimental versions to ensure that items were seen an even number of times in each condition across participants. Our within-participants and within-items counterbalanced design ensured that any interitem differences would be cancelled out across the two levels of contextual diversity. Participants were pseudorandomly and evenly assigned to one of the four experimental versions, there were exactly 25 participants in each version. The dependent measures were: spelling accuracy (Levenshtein distance) in word-form recall, accuracy in word-form recognition, and accuracy in recalling semantic features in word-meaning recall. Additionally, we conducted an exploratory analysis on the comprehension ratings of the word meanings.

### Procedure

The experiment was run online using Gorilla Experiment builder (www.gorilla.sc; Anwyl-Irvine et al., 2020). In order to try and mimic naturalistic word learning conditions in which learners are usually focused on comprehension and not on explicit memorization (Hulme et al., 2019), participants were told that the experiment was investigating how people understand new words: They were *not* told to remember the new meanings presented. Participants read the information sheet and gave their consent to take part, and then answered demographic questions. The experiment comprised three phases, completed in a single session: (1) training phase (paragraph reading; around 15 minutes), (2) filler task (Towers of Hanoi, around 2 minutes), (3) testing phase (word-form recall, word-form recognition, and word-meaning recall; around 13 minutes).

In the training phase participants were instructed to read a series of paragraphs carefully. They were told that after some paragraphs they would be asked questions to check their understanding and to rate their understanding. Participants each read a total of 40 paragraphs one at a time: five paragraphs for each of the eight pseudowords. Four of the sets of paragraphs presented items in the low contextual diversity condition and four presented items in the high contextual diversity condition, with the eight sets of paragraphs presented in a randomized order. After each set of five paragraphs, (to ensure attention) participants were asked a comprehension question that asked about details of the final paragraph they had just read (without probing details of the new word meanings). Participants were excluded if they got more than two questions wrong. Following the comprehension question for each item, participants were asked to rate their comprehension for that item, for example: *“How much did you understand about the new type of carrot that you read about in the previous paragraphs?”* Participants rated their comprehension on a 1–7 scale, where 1 indicated that they did not understand anything and 7 indicated that they had understood perfectly.

After the training phase, participants completed the Towers of Hanoi game to introduce a short (2-minute) delay to counteract any recency effects for items encountered at the end of the training session. The Towers of Hanoi game is a spatial task with no substantial linguistic components so should not produce direct linguistic interference with learning (which could have potentially affected some items more than others).

The test phase began with word-form recall: participants were asked to type into a text box each of the new words in response to prompts that included the base word (e.g., 'A type of carrot was called a …'). The instructions specified: “We will give points for every letter that is correct, so please try to guess the word even if you are unsure of your answer. If you really have no idea please type ‘don't know.’” The order of the items was randomized.

Participants then completed the word-form recognition test. The questions were identical to the word-form recall task, except that answers were in multiple-choice format. Participants were instructed to click on the correct word form, selecting from a list of all eight of the word forms they had been trained on. Participants were again asked to guess if they were unsure of the answer. The order of the questions was randomized and the order of the response options was randomized for each question.

Finally, in the word-meaning recall task, participants were asked to recall as much information as they could about the meanings of the newly learned words in response to a prompt that included both the base word and the novel word form (e.g., ‘Earlier you read about a type of carrot that was called a flam. Describe everything you learnt about a flam.’). The instructions emphasized that points would be given for every piece of information remembered, so participants were encouraged to write as much detail as possible and to try to guess if they were unsure of their answer; if they could not remember anything they were asked to write ‘don’t know.’ Although there were only five key semantic features for each item, seven numbered text boxes were provided for their response to encourage maximum recall. The items were presented in a randomized order.

At the end of the experiment participants were asked if they had written any notes about the paragraphs during the training task. Participants were asked to rate how difficult they found the experiment on a scale from 1 (*not difficult at all*) to 7 (*very difficult*). Finally, they were asked to state what they thought the main aim of the experiment was.

### Data coding and analysis procedure

For the word-form recall test, to provide a continuous measure of the similarity between a participant’s typed response and the correct spelling, Levenshtein distances were calculated using the *vwr* package (Version 0.3.0; Keuleers, 2013) in R (Version 4.0.0; R Core Team, 2020): lower Levenshtein distances indicate a more accurate response (0 indicates a fully correct response, ≥4 indicates that no letters were correctly recalled). ‘Don’t know’ responses were left blank so that the Levenshtein distance for these would be the total length of the word form (i.e., four letters). Responses for the word-form recognition test were coded as ‘1’ if the correct word form had been selected, or ‘0’ for incorrect.

Responses for the word-meaning recall test were manually scored as the number of semantic features correctly recalled (/5) for each item. Responses were leniently coded, with 1 point given for each correctly recalled feature, or 0 for incorrect or not recalled features. Responses were coded blind to condition, and Table 2 listing each of the five semantic features for each item was referred to during coding. Following a preregistered protocol, two researchers independently coded 10% of the data, which resulted in 95% coding consistency. Following discussion of the coding protocol a further 10% of the data were double coded, which again resulted in 95% coding consistency. Following further discussion to resolve inconsistencies, one of these researchers proceeded to code the remainder of the data.

Data for each dependent measure were analyzed separately using R (Version 4.0.0; R Core Team, 2020). Models were fitted using the packages *lme4* (Version 1.1-27.1; Bates et al., 2015) and *ordinal* (Version 2019.12-10; Christensen, 2019), and figures were made using *ggplot2* (Version 3.3.5; Wickham, 2016). A linear mixed effects model was used to analyze the Levenshtein distance data for the word-form recall test, and a logistic mixed effects model was used to analyze the binary accuracy data for the word-form recognition measure. Cumulative link mixed models were used to analyze the data for the word-meaning recall test and for the exploratory analysis of comprehension ratings. The contrast for the effect of contextual diversity was defined using deviation coding (high: −0.5, low: 0.5). Random effects structures were determined by identifying the maximal model (Barr et al., 2013), which included random intercepts and random slopes for the effect of contextual diversity by-participants and by-items. However, for all analyses the maximal model either failed to converge or resulted in a singular fit, indicating that the model was overparameterized (including when simplified as recommended by Barr et al., 2013). We therefore used a data-driven forward ‘best-path’ model selection approach (Barr et al., 2013) to identify the most complex model supported by the data. The final model random effects structure for all analyses included only random intercepts by-participants and by-items. Our analysis scripts and data files used for the analyses are available on the OSF (https://osf.io/2bnw3). Significance of the fixed effect of contextual diversity was determined using likelihood ratio tests comparing the full model to a model with the fixed factor of contextual diversity removed.

We supplemented our preregistered analyses with exploratory Bayes factor analyses (Wagenmakers, 2007) to assess the evidence for a null or significant effect of contextual diversity for each of our measures. Bayesian mixed effects models were fitted using the *brm()* function from the *brms* package (Version 2.16.3; Bürkner, 2017). These models had the same structure as the preregistered analysis models. We assumed noninformative priors for our fixed effect of contextual diversity: *normal(0, 1)*. All models had four chains and 12,000 iterations (with 2,000 warmup iterations), and model convergence was confirmed by consulting the Rhat statistic. We then used the *hypothesis()* function to compute the Bayes factor (*BF*_10_) for the fixed effect of contextual diversity for each model. We referred to the Jeffreys (1961) evidence classification scheme (with labels updated by Lee & Wagenmakers, 2013) in the interpretation of our Bayes factors.

## Results

### Word-form recall

**Fig. 1:**
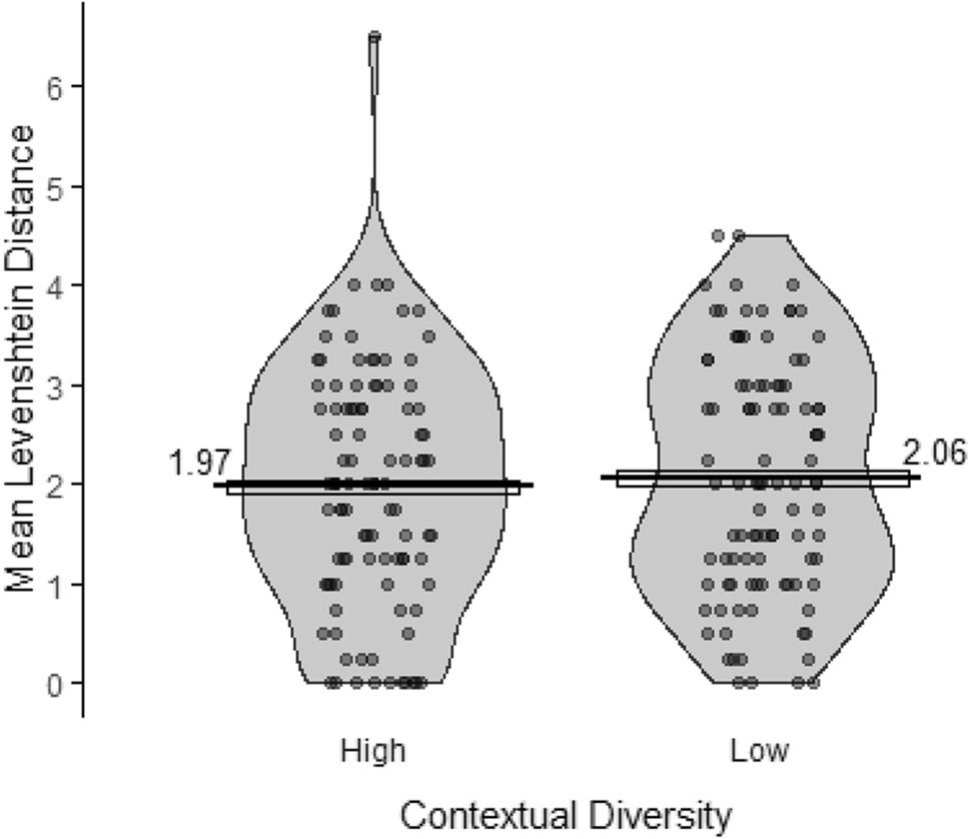
Mean Levenshtein distances between responses and correct spellings in the word-form recall test for the high and low contextual diversity conditions. Points represent participants’ condition means, the bars represent the mean across participants in that condition, the boxes around the means represent within-participant standard errors (Morey, 2008), and the violins represent the distribution of the data

The Levenshtein distances comparing participants’ responses to the correct spellings are shown in Fig. 1. Spelling accuracy was highly variable across participants and quite low on average (a mean score of around 2 indicates 2/4 letters correct, i.e. around 50% mean accuracy across both conditions).^4^^,^^5^ A model with the following structure was fitted to the Levenshtein distance data: *lmer(LevenshteinDistance ~ 1 + ContextualDiversity + (1|Participant.ID) + (1|Item)*. There was no significant difference between the high (*M* = 1.97; *SE* = 0.07) and the low (*M* = 2.06; *SE* = 0.07) contextual diversity conditions, *χ*^2^(1) = 0.83, *p* = .362. The Bayes factor indicated moderate evidence for a null effect of contextual diversity on word-form recall (*BF*_10_ = 0.14).

### Word-form recognition

**Fig. 2:**
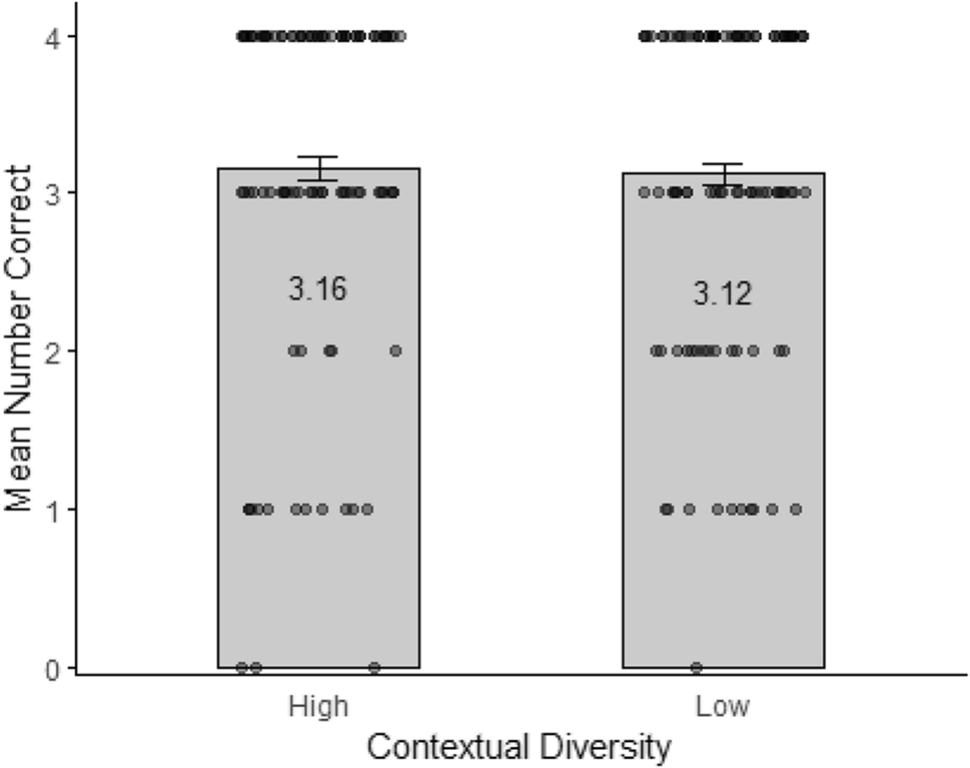
Mean number of correct responses in the word-form recognition test for the high and low contextual diversity conditions (max = 4 in each condition). Points represent participants’ number of correct responses in each condition, the grey bars represent the mean across participants in that condition, and the error bars represent within-participant standard errors (Morey, 2008)

The accuracy data for the word-form recognition task were analyzed using the following model: *glmer(Accuracy ~ 1 + ContextualDiversity + (1|Participant.ID) + (1|Item)*. Accuracy was high overall (Fig. 2) and there was no significant difference in accuracy between the high (*M* = 3.16; *SE* = 0.07) and the low (*M* = 3.12; *SE* = 0.07) contextual diversity conditions, *χ*^2^(1) = 0.28, *p* = .599. The Bayes factor indicated moderate evidence for a null effect of contextual diversity on word-form recognition (*BF*_10_ = 0.23).

### Word-meaning recall

**Fig. 3:**
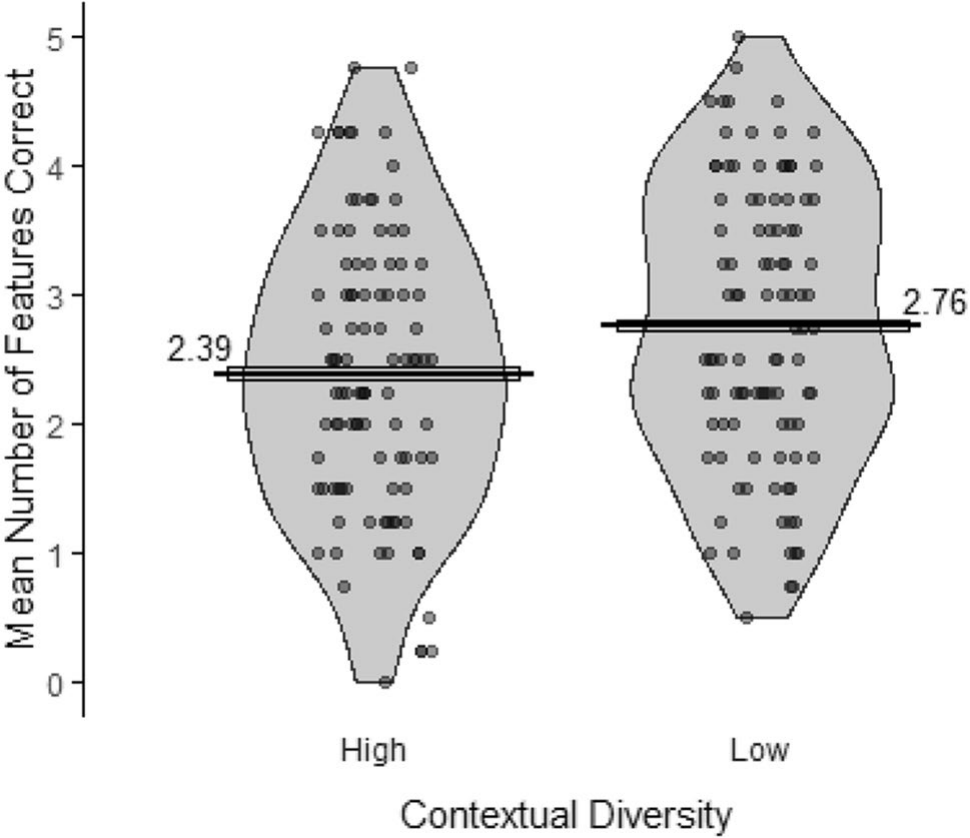
Mean number of features correctly recalled in the word-meaning recall test for the high and low contextual diversity conditions. Points represent participants’ condition means (max = 5), the bars represent the mean across participants in that condition, the boxes around the means represent within-participant standard errors (Morey, 2008), and the violins represent the distribution of the data

The word-meaning recall data were analyzed using the following model: *clmm(SemanticFeaturesScore ~ 1 + ContextualDiversity + (1|Participant.ID) + (1|Item)*. The number of semantic features correctly recalled (Fig. 3) was significantly higher for the low (*M* = 2.76; *SE* = 0.04) compared with the high (*M* = 2.39; *SE* = 0.04) contextual diversity condition, *χ*^2^(1) = 36.63, *p* < .001. The Bayes factor indicated extreme evidence in support of an effect of contextual diversity on word-meaning recall (*BF*_10_ > 100).

### Comprehension ratings

**Fig. 4:**
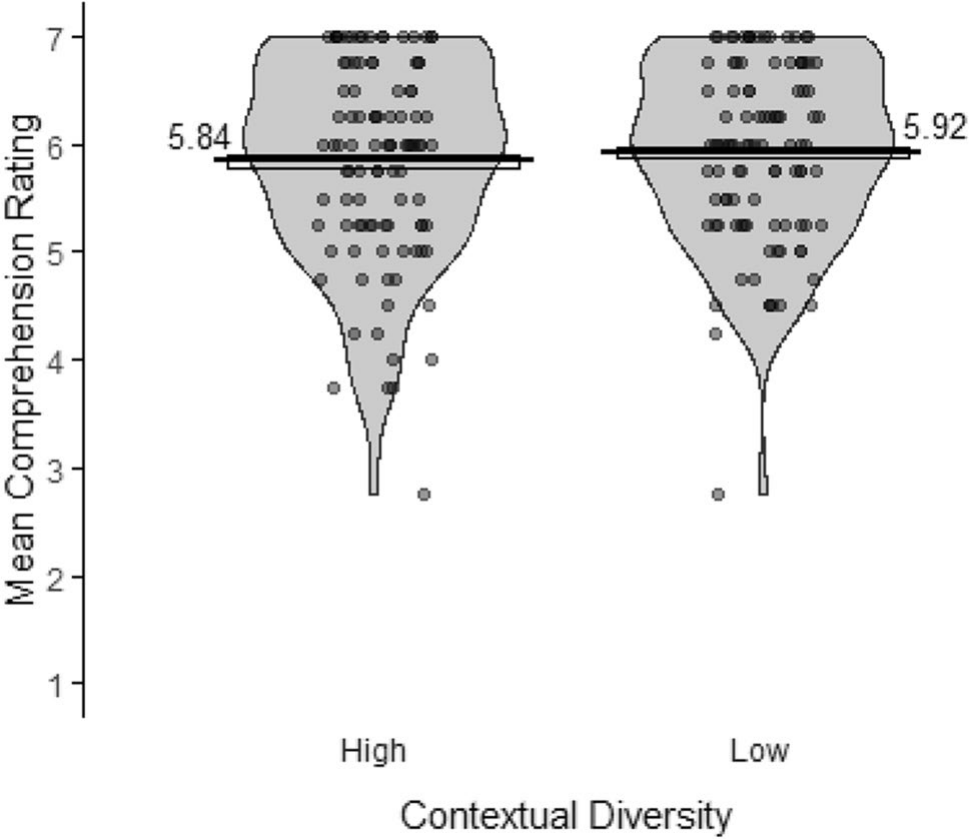
Mean comprehension ratings of the word meanings during paragraph reading (1 = *did not understand at all*; 7 = *understood perfectly*) for the high and low contextual diversity conditions. Points represent participants’ condition means, the bars represent the mean across participants in that condition, the boxes around the means represent within-participant standard errors (Morey, 2008), and the violins represent the distribution of the data

An exploratory analysis was conducted on the comprehension ratings for the word meanings during paragraph reading (Fig. 4). Note that this task was primarily included to encourage participants to focus on comprehending the narratives and not on explicit memorization strategies, and no predictions were made. Ratings were high across both conditions, indicating that participants found it relatively easy to extract the meanings of the novel words. A model with the following structure was fitted to the comprehension ratings: *clmm(ComprehensionRating ~ 1 + ContextualDiversity + (1|Participant.ID) + (1|Item)*. There was no significant difference between the high (*M* = 5.84; *SE* = 0.05) and the low (*M* = 5.92; *SE* = 0.05) contextual diversity conditions, *χ*^2^(1) = 0.67, *p* = .415. The Bayes factor indicated moderate evidence for a null effect of contextual diversity on comprehension ratings (*BF*_10_ = 0.20).

## Discussion

To investigate how situational (contextual) diversity affects the earliest stages of word learning via reading, in this preregistered experiment 100 adults learned pseudowords encountered within either a single coherent narrative context, or across several different narrative contexts. Unlike previous studies, the specific semantic features associated with the pseudowords were held constant across the diversity conditions, to ensure that any observed effects were not driven by differences in polysemy, which is well known to influence how easily words are learned and processed (Rodd, 2020, 2022).

Contrary to our predictions, contextual diversity did not influence word-form learning as assessed by both recall and recognition measures; Bayes factors indicated moderate evidence for these null effects. While this is consistent with some other studies (Bolger et al., 2008; Norman et al., 2022), others have found an effect of contextual diversity on word-form learning, be it beneficial (Frances et al., 2020; Johns et al., 2016) or detrimental (Mak et al., 2021). Performance was not at ceiling or floor: mean accuracy was around 2/4 letters spelled correctly in recall and 75% accuracy in recognition, albeit with considerable interindividual variability. This suggests that the tasks were sensitive to partial knowledge of the newly learned word forms. That said, some other studies used speeded measures of word-form recognition (Johns et al., 2016; Mak et al., 2021), which might have been more able to detect subtle effects of contextual diversity on word-form recognition efficiency (although see Norman et al., 2022). We also note that our measures of word-form learning were not purely orthographic as they also contained semantic elements. However, given that we observed a significant negative effect of diversity on word-meaning recall, it seems more likely that including semantic aspects in the measures of word-form learning might have increased the likelihood of observing an effect of diversity.

Unlike previous studies, we controlled for the variability in word meanings (i.e., polysemy) across diversity conditions. Previous findings may reflect a confound with polysemy. Polysemous words are processed more quickly in lexical decision tasks but show a processing disadvantage in semantic classification tasks (Hino et al., 2006; Rodd, 2020; Rodd et al., 2002, 2004). Thus, a confound with polysemy could potentially explain Johns et al.’s (2016) finding that words encountered in high contextual diversity conditions were recognized faster and more accurately. However, Mak et al. (2021) found the opposite pattern of results: words experienced in a single topic were initially recognized more accurately. Polysemy might exert a stronger influence on learning new word forms than situational (contextual) diversity. These differences could possibly explain why contextual diversity behaves differently across studies of word learning, and across different measures of learning (Bolger et al., 2008; Frances et al., 2020; Johns et al., 2016; Kachergis et al., 2009; Mak et al., 2021; Norman et al., 2022; cf. Brekelmans et al., 2022).

In contrast to the null effect on word-form learning, there was a clear negative effect of contextual diversity on learning word meanings. Significantly more semantic features were correctly recalled for words learned in a single narrative context (the Bayes factor indicated extreme evidence supporting this effect), consistent with some but not all previous work (Mak et al., 2021; Norman et al., 2022; cf.: Bolger et al., 2008; Frances et al., 2020; Johns et al., 2016; Kachergis et al., 2009). This finding fits with the general observation that across domains, variability typically makes initial acquisition more challenging (Raviv et al., 2022). We observed this effect of contextual (situational) diversity while controlling for polysemy (heterogeneity) and frequency of presentation. There was no difference in rated comprehensibility between the high- and low-diversity conditions. This contrasts with Johns et al. (2016) who found lower comprehension ratings for passages in which words were encountered in high-diversity conditions. Our findings indicate that participants had no additional difficulty in understanding the words in the high-diversity condition during reading, suggesting that the benefit of lower contextual diversity is likely a retention/memory effect.

This benefit for consistency in narrative context may reflect two different mechanisms, perhaps working in parallel. First, the coherent, low-diversity narratives will likely have required fewer cognitive resources to comprehend compared with the more disjointed high-diversity narratives. This reduction in processing load could may have allowed greater attention to be devoted to processing the new words, facilitating the anchoring of nascent knowledge (Franconeri et al., 2013; Mak et al., 2021).

In addition, the contextual diversity effect may more directly reflect the quality of the discourse representations that are built in the two conditions. The low-diversity condition allows readers to build a single coherent, and relatively enriched, narrative representation into which new words can become more tightly integrated, compared with the less coherent high-diversity condition in which the new words become (more weakly) linked to multiple different discourse representations. Discourse coherence could therefore be considered the antithesis of contextual diversity; relatedly a study by Sullivan et al. (2019) demonstrated that participants’ expectations about the coherence of a narrative can influence their interpretation and learning of novel words. It is possible for future studies to disentangle these effects by, for example, comparing cases where a particular to-be-learned word is always presented within a single narrative context, but where the internal coherence of this narrative is varied. A single well-integrated representation is likely to be better able to support subsequent retrieval (as measured by meaning recall) than multiple, weaker representations. This explanation, which assumes that the effect is driven by the quality of the learned semantic/discourse knowledge, can explain why diversity influenced word-meaning but not word-form learning.

This account is closely linked to recent proposals under which sentence comprehension gives rise to newly built (episodic) representations that bind together different discourse elements, and that these representations play an important role in supporting word learning (see Episodic Context Account; Curtis et al., 2022; Gaskell et al., 2019; Mak et al., 2022). We suggest that the current manipulation of contextual diversity has impacted the quality of this critical, temporary, discourse representation that may be important for retention of newly learned word meanings.

More broadly, this work contributes to a growing body of work that emphasizes the importance of prior knowledge in supporting word learning. Rodd (2020) emphasizes that word learning occurs within highly structured language system and that learning is significantly enhanced when the to-be-learned information shares informational content with existing knowledge. Previous work has focused on the beneficial impact of long-term semantic knowledge on word learning. For example, learning new meanings for familiar words is facilitated when new meanings can be anchored onto existing related knowledge about that word’s form (Maciejewski et al., 2020; Rodd et al., 2012). Similarly, Mak et al. (2021) found enhanced learning for novel words that were consistently linked with a single familiar topic (e.g., Brexit), which would have likely facilitated integration of the new words with the existing semantic knowledge. Our work extends these findings to show that word learning can be enhanced by consistently anchoring new words into *newly built* representations of the current discourse.

The current study necessarily focuses on one specific form of word learning, and this may limit the extent to which these findings generalize to other learning situations. First, in the current experiment participants were given a relatively explicit definition of the word’s meaning in their initial encounter. As previously discussed, although there are instances in natural word learning where such explicit definitions are provided, it is perhaps more often the case that learners must infer new word meanings from surrounding context. While the present study bridges the gap between more artificial learning paradigms (e.g., paired associate learning tasks; Jones et al., 2012), and more highly naturalistic paradigms in which words are learned incidentally through reading naturalistic narratives (e.g., Godfroid et al., 2017; Henderson et al., 2015; Hulme et al., 2019; Hulme & Rodd, 2021, 2023), future work should explore the impact of diversity on a range of experimental paradigms and stimulus types to more closely capture the full diversity of word learning situations.

Second, the present study used novel instances of already familiar concepts (e.g., a new variety of purple carrot) that were distinguished by five invented semantic features that constituted the new meaning. While some of these features correspond to a core part of the novel definition that allows participants to distinguish it from the base meaning (e.g., the purple colour of the “flam”), other properties are more incidental/optional (e.g., that ‘flams’ make good crisps). The latter properties might be considered to be ‘world knowledge’ rather than ‘word knowledge’ and such semantic facts may not be part of the lexical meaning for the novel word (Jackendoff, 2022), although there may be no principled boundary between these two constructs (Casasanto & Lupyan, 2015). We therefore considered any of the novel semantic information that could help readers distinguish the new lexical entries from other words with similar meanings as useful components of their lexical meanings. This dimension of semantic features of word meanings is potentially significant, as the various features of a word’s meaning could potentially be differently influenced by contextual diversity depending on whether they form a core or more incidental/optional part of a word’s meaning.

Finally, it is important to emphasize that the current study focuses on the earliest stages of word learning, suggesting that consistent anchoring of to-be-learned words to a single hub of existing knowledge can be beneficial to the initial encoding and retrieval of previously unfamiliar information. In contrast, studies of contextual diversity for familiar words have often found a processing benefit for words that occur in more diverse contexts (Adelman et al., 2006; Brysbaert & New, 2009; Hoffman et al., 2013; Johns et al., 2012, 2016; Jones et al., 2012; McDonald & Shillcock, 2001). Diversity effects therefore seem to vary across the time course of learning, whereby lower diversity is initially beneficial for anchoring nascent knowledge, and greater diversity may become more beneficial with increasing familiarity and context independence (Mak et al., 2021; Raviv et al., 2022).

### Supplementary Information

Below is the link to the electronic supplementary material.

Supplementary file1 (PDF 82.4 KB)

Supplementary file1 (PDF 391 KB)

## Footnotes

1 The other two forms of variability that they describe are ‘numerosity’ (learning from more vs. fewer examples) and ‘scheduling’ (learning under more vs. less varied practice schedules).

2 The pilot study used the same measures of word-form recognition and word-meaning recall, but did not include a word-form recall measure.

3 Stimuli were selected from this paper to facilitate future work looking at inferencing for novel words.

4 Levenshtein distances for most responses were in the range 0–4, but a small number of responses scored higher where a response included more than four letters.

5 An exploratory analysis confirmed that word-form recall performance (Levenshtein distance; high = poor performance) was moderately negatively correlated with word-meaning recall (number of semantic features correct; high = good performance) *r*(98) = −.44, *p* < .001.
