# Supplementary material for: Diversity of narrative context disrupts the early stage of learning the meanings of novel words
Source: Psychon Bull Rev. 2023 Jun 27;30(6):2338–50. doi: 10.3758/s13423-023-02316-z (PMC10728247; doi:10.3758/s13423-023-02316-z)
Supplement: Supplementary file 1 — Supplementary file1 (PDF 82.4 KB) [file 13423_2023_2316_MOESM1_ESM.pdf]

Table S1

Nonwords and brief description of their five corresponding scenarios.

Nonword	Scenario 1	Scenario 2	Scenario 3	Scenario 4	Scenario 5
Tock	Elderly couple with cold house	Manager of a furniture shop	Decorating apartment	Fitting in a new office	Family wins a home makeover
Lape	Family trip to the beach	Art project about nature	Arts and crafts videos on YouTube	Man loves collecting stationery	Mother buys daughter gift
Flam	Recent vegan	Supermarket chain selling produce	Mother feeding young daughter	Man with allotment of vegetables	Unhealthy person on a diet
Spea	Woman buying for a friend's birthday	Celebrity model doing a video campaign	Review of products	Woman's son ruining the product	Woman dressing up for her wedding
Clab	Couple who own a café	Woman sorting her wedding gift list	Woman has a friend staying over	Office buys appliances for their kitchen	Woman decluttering her kitchen
Barl	Woman hosting a dinner	Man heating food from fridge	Online writer for health blog	Man eating lunch on holiday	Farmers making profit recently
Tace	Commuter on a train	School student going to school	Teacher late for work	Person who worked for design team	Person reading a fashion magazine
Fisk	Man on career break abroad	University student at a bar	Woman created a drinks company	Cat caused a spill on the carpet	Woman hosting house party
Bamp	Baby with sensitive skin	Person has a cold	Plumber at a job	Advertisement on TV	Person reading social media post

Nonword	Scenario 1	Scenario 2	Scenario 3	Scenario 4	Scenario 5
Hoad	Couple looking to buy a pet	Woman going on holiday	Veterinary student doing research	Man going on a stroll	Fitness instructor going for a run
Coft	Man doing grocery shopping	Man walking home	Office worker on smoke break	Boy playing at home	Man has chest problems
Veak	Girl studying in biology class	Episode of a nature TV programme	Boy at grandma's house	Scientists studying genetics	Home infestation
Hust	Consultant at a conference	Air hostess's new uniform	First-time mum buying clothes	Eccentric secondary school teacher	Japanese fashion designer
Deam	Family on holiday at a cabin	Man part of design team	Employee storing in warehouse	School competition	Christmas presents
Zove	Friends on a road trip	Woman picking son up from school	Man stranded on mountainous road	Man going to work in rush hour	Car brand winning energy award
Yark	Picnic in the park	Show and tell event at school	Electricity cuts off at home	Boy playing with friends outside	Family going to grandparents' home
