# Supplementary material for: Diversity of narrative context disrupts the early stage of learning the meanings of novel words
Source: Psychon Bull Rev. 2023 Jun 27;30(6):2338–50. doi: 10.3758/s13423-023-02316-z (PMC10728247; doi:10.3758/s13423-023-02316-z)
Supplement: Supplementary file 2 — Supplementary file1 (PDF 391 KB) [file 13423_2023_2316_MOESM2_ESM.pdf]

Table S2

Paragraphs for all items in the high and low contextual diversity conditions.

Item	Set	Paragraph	High CD	Low CD
Tock	A	1	Meryl was 75 years old and healthy for her age. Her husband, Arthur, was a few years older than her and needed a little more help but they both got by fine. Meryl still managed to do her own cooking and cleaning but would occasionally get help from housekeeping or her children and grandchildren. The family regularly visited them and one day, her grandson Harry noticed that the house was fairly cold. He'd heard of a **tock** which was a type of window blind *that could both remove or trap heat inside*.	Meryl was 75 years old and healthy for her age. Her husband, Arthur, was a few years older than her and needed a little more help but they both got by fine. Meryl still managed to do her own cooking and cleaning but would occasionally get help from housekeeping or her children and grandchildren. The family regularly visited them and one day, her grandson Harry noticed that the house was fairly cold. He'd heard of a **tock** which was a type of window blind *that could both remove or trap heat inside*.
		2	Aaron was a manager at a home furnishings retailer. He initially started off as a store assistant and soon became the store supervisor. Aaron was confident in what he did, and he had great product knowledge so he was quickly promoted to a managerial role. Achieving sales targets was really important for the company because they were an independent store that solely relied on in-store purchases. The newest product that came in was called a **tock** and *they cost thousands of pounds each*.	Meryl thought that this winter was especially cold. The air felt very crisp and the nights were very chilly. She liked to keep warm in the evening with some tea and biscuits and watch TV in her pyjamas and fluffy robe. Meryl's daughter bought her and Arthur matching robes last Christmas and she loved them. The next day, the **tock** her grandson Harry had ordered arrived and the workers fitted them on. She saw on the invoice paper that *it cost thousands of pounds*.

Item	Set	Paragraph	High CD	Low CD
		3	Millie recently purchased a one-bedroom apartment. The location was perfect because it had ideal travel connections, great restaurants and many things to do nearby. She was really looking forward to living there so she decided to move in immediately with temporary furniture instead of taking up her friend's offer to stay at her home. Millie was a perfectionist and she wanted the interior design to be absolutely flawless. For example, she bought a new **tock** *which was made to measure so it fit perfectly on her kitchen window*.	Every Tuesday afternoon, Meryl and Arthur walked to the local community centre where elderly people would get together to socialise, take part in activities, and eat lunch. They had been going for years now and really enjoyed it because they met such great people. It was really nice to get out of the house and sometimes the centre would organise days out. One day, Meryl mentioned to her friend Agnes that her grandson had bought her a new **tock**. She told Agnes that *it was made to measure so it fit perfectly on her kitchen windows*.
		4	The company Calvin worked for moved office locations last week. It wasn't so much of a problem for Calvin because he still only needed to take one train to get there. However, he knew that for some of his colleagues it would be quite a trek. On Monday he began settling into his new desk. There was much more open space than in the previous office. However, direct sunlight seemed to be hitting his eyes and he thought a **tock** *would be useful because they are transparent in the day*.	Both Meryl and Arthur were into gardening and the cold winter didn't stop either of them. This winter Meryl decided to grow asparagus, onions, and broad beans. It was all growing very well, and they were looking forward to cooking with it. Meryl liked to invite her children and grandchildren for home-cooked meals made using their produce. Meryl didn't always need to step outside to have a look at the vegetables, she could look through the **tock** *which was transparent in the day*.
		5	Mason and Sophia were the parents of four children. Mason recently got a new job as an IT support officer in the same school that Sophia worked as a teaching assistant. Sophia was such a hard worker and was	Meryl and Arthur invited everyone over for lunch on Sunday. Meryl's daughter Betty came over earlier than the rest and together they prepared a Sunday roast with roast chicken, roast potatoes, Yorkshire puddings,

Item	Set	Paragraph	High CD	Low CD
			constantly busy, so she didn't have much time for herself. One day, Mason applied for a famous home makeover television show to surprise Sophia and it was accepted. Sophia was over the moon and she loved absolutely everything, especially the **tock** *which was button-operated*.	and of course the garden vegetables. Meryl and Arthur loved the atmosphere when it was a full house. The youngest grandchild was almost three years old and he liked touching everything in the house. He started messing about with the **tock** *by pressing the buttons as it is button-operated*.
Lape	A	1	Isabella and her family were on their way to Southend beach. Her and her younger brother Noah sat at the back of the car and occupied themselves with things to do. Noah loved handheld gaming consoles, so he spent the entire car journey playing video games. Isabella brought her iPod and headphones and listened to music, mainly pop songs. Isabella loved to draw and colour, so she packed her sketchbook and her new set of **lape**. These were a type of crayon that were *eco-friendly because they were biodegradable*.	Isabella and her family were on their way to Southend beach. Her and her younger brother Noah sat at the back of the car and occupied themselves with things to do. Noah loved handheld gaming consoles, so he spent the entire car journey playing video games. Isabella brought her iPod and headphones and listened to music, mainly pop songs. Isabella loved to draw and colour, so she packed her sketchbook and her new set of **lape**. These were a type of crayon that were *eco-friendly because they were biodegradable*.
		2	Ethan was a primary school student in Year 4. He really loved football and was in a local football club. He went to training sessions three times a week and his skills were rapidly improving. His training coach was very impressed by his progress. Ethan's parents were also very happy that he was doing well in school. His teacher had asked the children to do an art project about nature over the weekend. Ethan decided he would	There was still about an hour left of the journey, so Isabella decided to get out her sketchbook and draw. She would always draw and doodle, even in her school lessons if they were particularly boring. While in the car, Isabella thought about the time she and her school friend were caught doodling on their desks in class and sent to the headteacher's office. Isabella remembered that they had used her friend's **lape** to do the

Item	Set	Paragraph	High CD	Low CD
			draw a forest with his new set of **lape** because *they were fully erasable so helpful in case he made any mistakes*.	doodling. *It is fully erasable, so they promised the headteacher they would clean it up*.
		3	Madison was 19 years old and in college. Last summer, she was really bored at home so she decided to start up a YouTube channel. She made all kinds of videos from arts and crafts to video blogs about her day. She saw that her subscriber count was gradually increasing, and she was really happy, but she needed to be more consistent with uploading. One of her subscribers requested that she draw a portrait of herself using a **lape**. She ordered it online to do so and interestingly, *she saw that it was made from plant-based wax*.	The family finally parked their car and headed up to the beach. It was surprisingly quiet for a weekend but probably because it wasn't the sunniest day. The family didn't mind because they visited Southend often and it was nice to generally spend time near the beach. Isabella's mum set out the beach towels and the snacks she had packed. Isabella sat on the side and brought out her sketchbook and tools. She wanted to draw a picture of the view ahead with her set of **lape**. *Interestingly, they are made from plant-based wax*.
		4	Sebastian studied English Literature at university and was really passionate about books and writing. His dream was to become a successful author of fictional books. Sebastian had written a few short books and published them online. One of his books had over 100 reads. Sebastian was also really into collecting stationery. He had a study room in his home and one wall was dedicated to stationery. His newest purchase was a set of **lape** *which can be used for drawing on all surfaces*.	It was nearing 1 o'clock and the family were beginning to get hungry—they hadn't packed any lunch from home. Isabella's dad had been craving fish and chips for the longest time, so suggested they eat it on the beach. Isabella went with her dad to one of the fish and chip shops and helped him bring back the food. Once lunch was over, Isabella resumed her drawing. It was turning out very nice and she was pleased. She liked using a **lape** *because it can be used for drawing on all surfaces*.

Item	Set	Paragraph	High CD	Low CD
		5	Allison worked as Human Resources officer in a corporate organisation. Her job was interesting because she dealt with interesting cases and met people from all different levels and backgrounds. Allison asked her manager if she could leave work early on Friday because her daughter had performed really well in her Year 6 SATs and she wanted to take her out. Her daughter loved drawing and colouring, so she bought her a set of new **lape** *which had different fruit scents according to the colour*.	Some more time had gone by and the temperature had dropped slightly. Isabella's mum told them they should leave soon. The sky seemed to be turning grey and she didn't want them to get caught in any rain. Noah was disappointed because he wanted to spend more time at the beach, but their dad reassured him they would come back during the school holidays. Isabella's picture of the beach was finally finished, and she showed her family. She loved that each **lape** *had a different fruit scent according to the colour*.
Flam	A	1	Jane had just become a vegan. She was trying out loads of new foods to make up for cutting out so many of her usual ingredients. For her, one of the big advantages of making the decision to become vegan was that her diet was actually more varied than it had been before. And it seemed to be really easy to get hold of unusual ingredients these days. One of her favourite finds was a **flam** – an unusual type of *purple* <u>carrot</u>.	Jane had just become a vegan. She was trying out loads of new foods to make up for cutting out so many of her usual ingredients. For her, one of the big advantages of making the decision to become vegan was that her diet was actually more varied than it had been before. And it seemed to be really easy to get hold of unusual ingredients these days. One of her favourite finds was a **flam** – an unusual type of *purple* <u>carrot</u>.
		2	The supermarket was trying to encourage buyers to start buying some of their more unusual foods for the first time. They had started putting up information signs telling shoppers all about some of these products. For example, the sign about **flam** had lots of information about its health benefits – it emphasised that *they were a good source of*	Jane had started reading a lot more about the health benefits of different foods. She found it really interesting. And there was so much to read – it seemed that every day scientists were discovering new information. For example, she had recently learned that **flam** had lots of health benefits – the article she had read emphasised that *they were a good*

Item	Set	Paragraph	High CD	Low CD
			vitamins. Time would tell whether this new approach would actually change people's shopping habits.	*source of vitamins*. She was really making an effort to make her diet healthier, but it was hard to keep up with all the information.
		3	Kerry's daughter was now two years old. She was trying to get the toddler to eat a more varied diet. She'd recently discovered vegetable crisps, which were a good way of introducing new flavours. She had found *you could even get crisps made of* **flam**. Her daughter really liked them. Kerry was determined that she wouldn't end up with a fussy eater. It must be a nightmare to have to feed kids who would only eat certain foods.	One of the things that Jane was eating more of since she became vegan was vegetable crisps. They were a convenient snack to have in her work bag. They came in lots of different varieties – she had found *you could even get crisps made of* **flam**. She really liked them. Jane was glad that she wasn't a fussy eater. It must be a nightmare to be a vegan if you would only eat certain fruit or vegetables.
		4	Craig had an allotment where he grew vegetables. The allotment was about a 10-minute walk from his house and he tried to spend some time there after work most evenings in the summer. He was a generous guy and shared the vegetables he grew with his friends and family. He was always keen to try new varieties and had recently learned that **flam** *grew well in the UK climate* and so had decided to include them in his next cycle of vegetables.	As well as being vegan, Jane was also trying to save 'food miles' by buying as much locally produced food as possible and keeping an eye on what was currently in season in the UK. She often shopped at a small, local supermarket that stocked a lot of local produce. She had recently learned that **flam** *grew well in the UK climate*. Other foods were harder, especially fruit which often had a short season. It could be quite restrictive to only eat fruit that was currently in season in the UK.
		5	Elizabeth was on a diet. Again. Her diet was really unhealthy and something needed to change. She was keeping track of the calories in absolutely everything that she	Since becoming vegan, Jane hadn't had to worry so much about how many calories she ate. She'd found that cutting out meat and dairy had hugely reduced the amount of

Item	Set	Paragraph	High CD	Low CD
			ate. Some things had really surprised her. Like **flam**. Apparently, *they were really high in calories because they were so sweet and contained a lot of natural sugar*. There were lots of better options though and she was trying really hard not to even have high calorie foods in the house as she would find them hard to resist.	unhealthy food that she ate day-to-day. But the calorie content of some foods surprised her. Like **flam**. Apparently, *they were really high in calories because they were so sweet and contained a lot of natural sugar*. But that didn't stop her eating them. The rest of her diet was so healthy now.
Spea	A	1	Michelle had a busy weekend coming up. She went through the checklist on her phone and realised she had not yet bought a gift for her friend Daniella's 25th birthday party which was the next day. Daniella was a person who had almost everything, so Michelle really had to give thought to her gift. She knew Daniella liked makeup and heard about a new product called **spea**. This was a type of lipstick *that could be personalised* and lucky for Michelle, the company could offer same-day personalisation with her name.	Michelle had a busy weekend coming up. She went through the checklist on her phone and realised she had not yet bought a gift for her friend Daniella's 25th birthday party which was the next day. Daniella was a person who had almost everything, so Michelle really had to give thought to her gift. She knew Daniella liked makeup and heard about a new product called **spea**. This was a type of lipstick *that could be personalised* and lucky for Michelle, the company could offer same-day personalisation with her name.
		2	Jennifer was a celebrity model. Her schedule was always busy, and she found herself travelling all over the globe. Recently, her manager signed a contract with a designer makeup brand and she travelled to Los Angeles for the main video campaign. She would be shooting for eight hours with various makeup looks and products. Jennifer told her makeup artist she would like to use	Michelle travelled to the shopping centre and located the make-up store on her way in. All of a sudden, she heard her name being called and turned around to find her sister. Funnily enough, she was just thinking about her. They had a quick catch-up over coffee as Michelle explained she needed to go get Daniella a gift before the store closed. After entering the store, a friendly employee

Item	Set	Paragraph	High CD	Low CD
			a **spea** *because it comes with a lip scrub which is attached to the bottom of the casing*. She wanted to keep her lips soft and healthy throughout the day.	assisted Michelle and explained how the **spea** *comes with a lip scrub which is attached to the bottom of the casing*.
		3	Rosie was a young social media blogger who reviewed health, beauty and fashion products. She'd only started her page four months ago but saw her number of followers was steadily growing. Rosie really wanted to make this a success and liked to involve her followers at every stage. Many of her followers requested a review of a **spea**. She visited the company website and saw *it came in four different sizes from mini to large, she opted for the medium size*.	To keep her options open, Michelle explored the rest of the store's products. She noticed another customer looking at another brand which offered a bigger range of products. Michelle started to debate which Daniella would make the most use of and decided to stick with her first idea. But now, she was slightly confused because the **spea** *came in four different sizes from mini to large. Eventually, she went for the safest option and opted for the medium size*.
		4	Gemma's son was two and he was a handful. Most of her day consisted of keeping an eye on him as he liked to cause chaos. Gemma left her eldest daughter, Emily who was nine years old, in charge of him whilst she had a quick shower. However, Emily became distracted by her favourite television programme. So, when Gemma returned to her bedroom, she found her handbag tipped out and her son had broken her **spea**. She was upset because *it was really expensive*.	The store was nearing closing time and the queue was extremely long. Michelle just wanted to get home but the customer at the till was shouting at the staff and was causing a scene. She was charged twice for one of the items and was unhappy with the way the staff dealt with the mistake. Michelle finally reached the till to pay, but she had not checked the price, so it was not until then she realised the **spea** *was really expensive*. By this point, she was fed up and was willing to pay anything.
		5	It was the day of Mia's wedding. She had been planning the event months in advance	Michelle managed to complete a few other errands while she was in the shopping

Item	Set	Paragraph	High CD	Low CD
			and really wanted the day go to well. She organised the catering, the photography, her dress, and everything else alone. Mia was particularly anxious about the way her makeup would turn out. She wanted her makeup to be long-lasting and not dry after eating and drinking. She decided to use a **spea** on her lips *as it contained different moisturising oils*.	centre. But walking around for many hours was exhausting. She was ready to head home and call it a day. As she sat in the taxi, Michelle remembered the flyer the store assistant handed to her on her way out. It stated that the **spea** was beneficial for the lips as *it contained different moisturising oils*. She considered buying the product for herself but would await Daniella's review.
Clab	A	1	Liam and Charlotte had been happily married for 25 years. In the first year of their marriage, they moved away from the busy city to a smaller town in the UK. Twenty years ago, the couple opened their own café which quickly became popular among both locals and tourists. Liam recently bought a new **clab** for the café. It was a type of <u>toaster</u> *that came with an additional attachment to fry eggs*. He thought it would speed up the process when making breakfast for customers.	Liam and Charlotte had been happily married for 25 years. In the first year of their marriage, they moved away from the busy city to a smaller town in the UK. Twenty years ago, the couple opened their own café which quickly became popular among both locals and tourists. Liam recently bought a new **clab** for the café. It was a type of <u>toaster</u> *that came with an additional attachment to fry eggs*. He thought it would speed up the process when making breakfast for customers.
		2	Kylie's wedding day was quickly approaching. There was a very large budget and she had been organising the wedding for over a year. Her husband-to-be, Ethan, let Kylie take lead because he knew she loved to organise events. He thought she was a great wedding planner. Kylie set up a wedding gift list online, so guests could bring certain presents. One of the	The café opened at 7am every weekday and 9am every weekend. The couple knew almost all their customers as the majority had been regular customers for years. Every day was different, and they looked forward to the conversations they would have with people. In fact, Liam met one of his best friends at the counter many years ago. It was Saturday, and the first order of the day was

Item	Set	Paragraph	High CD	Low CD
			suggestions was a **clab** for their new home. *It had a flap at the bottom to directly release toast onto a plate*. She liked functional things.	beans on toast and Charlotte popped some bread into the **clab**. *It had a flap at the bottom to directly release toast onto the plate*.
		3	Bailey's friend, Alyssa, was staying over at her house. They hadn't seen each other in months because Alyssa lived in the opposite side of the country. Bailey was really sad when Alyssa moved but she was also happy because she was starting her dream job as a designer. Alyssa arrived late in the evening and she was tired so headed straight to bed in the guest room. In the morning, Bailey was making breakfast and she showed Alyssa her new **clab**—*it had a compartment where you could dollop spreads and jams to be spread for you*.	Orders began flying in. The beginning of the day was the busiest and Charlotte generally felt like she had no time to stop. Linda was the café's barista and she helped out a lot in making hot beverages and managing orders when Liam was not at the counter. Both Liam and Charlotte usually loved it when the café was busy because they liked to be occupied. Charlotte was enjoying using the **clab**—*it had a compartment where you could dollop spreads and jams to be spread for you*.
		4	Ian worked as a receptionist for an energy company in the City of London. He was in charge of welcoming visitors, answering queries, and maintaining security. He enjoyed his role, so he wasn't thinking about leaving any time soon. Ian was asked to order new appliances for the office kitchen because it had been recently renovated. He ordered the products online and he was especially happy with his purchase of a **clab** because *it was self-cleaning* which meant the crumbs could be easily removed.	The morning buzz had just calmed down, but it was already nearing lunch time and they would have to start preparing for the next wave of customers. Benjamin was one of the café's regular customers. He came to the café at 10am every day and stayed for a few hours, drinking numerous cups of black coffee. On the weekends, he liked to order a full English breakfast. While the café was quiet, Charlotte started to do a little cleaning. She noticed that the **clab** *was self-cleaning* which meant the crumbs could be easily removed.

Item	Set	Paragraph	High CD	Low CD
		5	Lucy had taken a week off work. She hadn't been on annual leave for a while so she felt she deserved some time off. But Lucy was a person who loved being busy and on her feet, so she looked around her apartment for things to do. Lucy decided to declutter her kitchen and then buy some new things because some of kitchenware and kitchen appliances were wearing out. She saw a **clab** in the kitchen appliances section of a furniture retailer. *It was very small and compact*, so she thought it would be useful for saving space.	Liam and Charlotte's daughter, Ava, was helping out in the café. She was 20 years old and home for Christmas. The café was fairly small so when it was busy, the front of the shop could get slightly cramped and Ava had to squeeze her way through to serve customers. But it had quietened down considerably as it was almost closing time, so everyone began tidying up the café. Ava noticed how the **clab** *was very small and compact*, so she thought it would be useful for saving space.
Barl	A	1	It was early morning on Saturday and Kate was shopping for food in her local supermarket. She was hosting a gathering with relatives at her house in the evening. Kate was unsure about what dishes she would make but she remembered her cousin John really enjoyed steak. In the ingredients aisle, Kate came across **barl** – this was a type of <u>ground pepper</u> which was mainly used in high-end restaurants because *it was expensive*. She had never seen this before.	It was early morning on Saturday and Kate was shopping for food in her local supermarket. She was hosting a gathering with relatives at her house in the evening. Kate was unsure about what dishes she would make but she remembered her cousin John really enjoyed steaks. In the ingredients aisle, Kate came across **barl** – this was a type of <u>ground pepper</u> which was mainly used in high-end restaurants because *it was expensive*. She had never seen this before.
		2	Richard had just come home from the gym. His wife was spending the evening with her friends, so she left him a note on the fridge that she'd prepared a plate of food that needed reheating. Richard was attempting a	There was a lot to do before Kate's relatives would start arriving. She asked her children to begin tidying up the house—starting with their bedrooms which were messy as usual. Kate found herself always reminding them

Item	Set	Paragraph	High CD	Low CD
			pescatarian diet, and it was going well so far. While he was waiting for his food to be heated, he picked up a jar of **barl** from the kitchen counter. *It had a very strong smell* which he was not expecting.	to make their beds. In the meantime, she began prepping the ingredients for dinner. She brought the **barl** close to her nose. *It had a very strong smell* which she was not expecting. It reminded her of something she'd smelled before, but she couldn't remember what it was.
		3	Tracey was an online writer for a health and wellness blog. She was tasked with writing the Wednesday article and had been doing so for the past six months. Tracey really enjoyed this as a freelance role alongside her full-time position as a nutritionist in a private practice. Her next piece was about **barl**. She was especially interested by *its distinct red colour*. Hopefully, the editor would find the piece worth publishing.	Cooking was in full swing and Kate was managing her time very well. She had prepared the starters, the main dishes, and even the desserts, and all that was left was the final touches. Kate was really impressed by the way her steak dish turned out. She was looking forward to using the **barl** as the finishing touch as *it had a distinct red colour*. But Kate was mostly looking forward to seeing her relatives as the last time they gathered was at Christmas.
		4	Steven was on holiday for two weeks for his best friend's stag do. They went to a restaurant for lunch and chose the table with a view of the sea. The sun was at its peak, and it was really warm, so Steven ordered a mojito to cool him down. He finished his entire plate of food and even ordered seconds. *The flavour of the* **barl** *that he ground on top was both sweet and spicy*. But not long after, he ended up feeling nauseous because he overate.	The guests finally arrived, and the adults were chatting while the children were playing. Kate was talking to her aunt Caroline who would soon be retiring and moving to Spain. Kate kept her eyes on the time because she planned to serve dinner at 8 o'clock. As everybody sat around the table, Kate brought over the food. She ground some **barl** over the plates. *The flavour of it was both sweet and spicy* and went well with the rest of the food.

Item	Set	Paragraph	High CD	Low CD
		5	Eduardo came from a family of farmers. His family had recently acquired many more acres of land. They initially began with tending to livestock such as cows and sheep. However, Eduardo himself became more interested in producing spices and seasonings, and eventually this became his main source of income. The majority of his portion of farmland was dedicated to making **barl**. *This was exclusively made in Brazil due to its hot climate* and Eduardo was making significant profits with demand rapidly increasing.	As the end of the night approached, Kate's relatives were slowly leaving. Although she really enjoyed the evening, Kate was exhausted and plumped down on the sofa to take a minute before she needed to get the children to bed and start tidying up. She had put all the dishes in the dishwasher, wiped the countertops and emptied the bins. While doing so, Kate came across the plastic packaging of the **barl** *and noticed it was exclusively made in Brazil, due to its hot climate*. She would definitely be buying this again.
Tace	A	1	Fred was a commuter. He travelled to his job as a lawyer in London each day on the train. He had been commuting for ten years and was beginning to struggle with so much of his time being spent on the train. Maybe he would soon find a job closer to home. He had recently bought himself a **tace** – a new type of umbrella that was *completely collapsible and packed away into the size of a glasses case* when he wasn't using it.	Fred was a commuter. He travelled to his job as a lawyer in London each day on the train. He had been commuting for ten years and was beginning to struggle with so much of his time being spent on the train. Maybe he would soon find a job closer to home. He had recently bought himself a **tace** – a new type of umbrella that was *completely collapsible and packed away into the size of a glasses case* when he wasn't using it.
		2	Katie had just started secondary school. Her parents had finally agreed to let her start walking to school each day. She was really excited about walking home. They had agreed the best route that avoided the main road and would only take her about 10 minutes to get home. They had even bought	Fred took the same train route home every day. And he always took the same leather bag on the train with him. It was a bit like a conventional briefcase, but not quite so rigid, and it had lots of pockets. The main section of the bag contained his laptop and all his work papers. He kept his **tace** in the side

Item	Set	Paragraph	High CD	Low CD
			her a new **tace**, which was *completely made of plastic – it had no metal components.* She kept it in her school bag so that she had it with her whenever she needed it.	pocket of this bag. It was *completely made of plastic – it had no metal components.* One time he left his bag on the train when he got off at his home station. Boy did he panic!
		3	Robert was a teacher. He was late for work. He had an important meeting and it was really important that he was on time that day. He had been ready to leave the house on time that morning–for once the kids had got themselves ready without too much nagging. It was his fault that he was late—he couldn't find his new **tace**, which he had only recently bought. *They were surprisingly cheap to buy,* and he was so pleased with it so he had taken it to show to his friend the night before and he couldn't remember what he had done with it.	Because Fred was so busy at work and with his long commute, he did most of his shopping online. The Wi-Fi on the new trains on his route was pretty good so he could get most of it done on his way home. And he always had his laptop with him on the train. He had bought his **tace** from Amazon. *They were surprisingly cheap to buy,* and he was so pleased with it, so he had had shown it to a few of his fellow commuters on the train. He'd made quite a few friends on the train–people who took the same train each day.
		4	Susie worked for a design company in Leeds. Her background was in marketing, but this job was the first time she had been involved in the design stage of the process. She was really enjoying working for the new company. She was part of the design team responsible for the **tace**. The team had won an innovation award because *it cannot get turned inside out by the wind.* She and the rest of the team were off to an award ceremony in Manchester. She'd not been on a night out since her son had been born.	Fred usually listened to podcasts on the train. It was good to have some light relief at the end of a busy day. But occasionally when he had nothing better to do, he'd end up reading the free magazines that were given out at the station near his work. The articles were mostly pretty boring, but he had recently read an interesting article about the design team responsible for the **tace**. Apparently, they had won an innovation award because *it cannot get turned inside out by the wind.* Fred was pleased to see them being rewarded for their work.

Item	Set	Paragraph	High CD	Low CD
		5	Karen was reading a fashion magazine while waiting for the hairdresser. It was annoying that the salon wasn't running on time as she needed to get back to work as soon as possible. Amongst all the usual magazine articles on up-market clothing and accessories, there was a piece on the **tace**, which was a very *recent invention.* It was unusual for this magazine to cover such topics and although she had no intention of buying one, she did find the article quite interesting.	Fred had recently started listening to a podcast where they discussed all the latest inventions and rated whether they were likely to be a success. Fred tried to remember the things they rated highly–people were always telling him he was a nightmare to buy presents for! Interestingly they did a feature on the **tace**. Fred hadn't realised that it was such a *recent invention.* It was definitely one of his better buys. It had encouraged him to start looking around for other cool gadgets.
Fisk	A	1	Anthony had been a stock trader for a large investment bank for almost 15 years. The early mornings and late nights were catching up to him and he needed a well-deserved break. Last month, Anthony's manager accepted his request for a 12-month career break and Anthony was overjoyed—he hoped to travel the world. The first destination he booked was in Southeast Asia. On the flight, he ordered a **fisk** – a type of beer. He noticed *the can was made of recyclable metal*.	Anthony had been a stock trader for a large investment bank for almost 15 years. The early mornings and late nights were catching up to him and he needed a well-deserved break. Last month, Anthony's manager accepted his request for a 12-month career break and Anthony was overjoyed—he hoped to travel the world. The first destination he booked was in Southeast Asia. On the flight, he ordered a **fisk** – a type of beer. He noticed *the can was made of recyclable metal*.
		2	Daniel was a first-year undergraduate student studying Physics at a prestigious university. It was the first time he was living away from home, but he was really enjoying it so far. Daniel was getting on with his responsibilities which included things he	It was two weeks into Anthony's career break and he was having the time of his life. He enjoyed being in his own company and immersing himself into the culture. Anthony budgeted his finances because he needed to be strategic about his spending costs. He

Item	Set	Paragraph	High CD	Low CD
			previously relied on his mother for i.e. cooking and cleaning! He spent most Friday and Saturday evenings with his friends at a local bar near his student accommodation and bought a **fisk** each time. *It was incredibly cheap*—a bonus for a budgeting student.	would stick to 3-star hotels, but this wasn't a problem—he wasn't looking for luxury living. Anthony was confident he would manage, as he would be mostly spending on food. He thought at least a can of **fisk** was *incredibly cheap*.
		3	Emma always wanted to be an entrepreneur. She liked to seek out business opportunities, from running a lemonade stall every summer to organising neighbourhood garage sales. By the time she was 18, Emma wanted to start up her own drinks company. She got together with her friend and did exactly that. They were soon making outstanding sales, but she realised she could make her main product, **fisk**, more profitable by *producing in Thailand*. The expenses were lower abroad.	Anthony visited all the tourist attractions and more—there was so much to see and do. He took advantage of the free shuttle service his hotel was offering and left early each day to explore the city centre and surrounding areas. Anthony visited new restaurants every day for lunch and loved the cuisine. There was one restaurant in particular with a very friendly waiter who spoke excellent English. He told Anthony that **fisk** *was produced in Thailand*.
		4	It was a rainy day and Jane was in the laundrette. She would normally spend time visiting the shops on the street while she waited, but she didn't fancy getting wet in the rain. Jane picked up a newspaper and read the front page. It was a story about the British royal family. When Jane finished reading, the machine was done. She really hoped the rug she'd put in would come out clean. Earlier, her cat had jumped onto the kitchen counter and knocked a can of **fisk**	One evening, Anthony was walking back to his hotel. He quickly became familiar with the local roads and hardly needed to use the maps on his phone. Also, the local people were so helpful if he needed directions or assistance anyway. He spotted a small grocery on the corner of the road and thought he would pick up a few snacks. The store owner appeared to be mopping up some mess a customer had made. The

Item	Set	Paragraph	High CD	Low CD
			onto the rug. *It is very fizzy* and the liquid spread everywhere.	customer accidently shook then opened a can of **fisk**, *not realising that it is very fizzy*.
		5	Natasha was hosting a Halloween party in the evening. She spent two whole hours getting dressed up as witch. Natasha was really excited to see what the others would be dressed as, especially her friend Adrian. Last year, he was the best zombie she had ever seen and even won the 'Best Costume' award. There was half an hour before people would start arriving, so Natasha began preparing the food and drinks. She bought many cans of **fisk** as she really loved its *unique pineapple flavour*.	Anthony was in a taxi headed for the airport and reflected on the first few weeks of his career break. He enjoyed every second and hoped the rest of the countries he would be visiting would be just as fantastic, or even more so. Checking in went smoothly and to Anthony's surprise, the airline offered him a free upgrade to business class. While waiting to board his plane, Anthony ordered a **fisk**, thinking of the first time he tried it. He loved its *unique pineapple flavour*.
Bamp	B	1	Ruth was a mother of three. Her first two children were in the early years of primary school and her third baby was now two months old. She was coping well with the additional responsibilities. But Ruth was so used to taking care of her older children that she almost forgot how different a baby's needs are. Ruth was on the look for new baby products and she heard about **bamp** – a new type of tissue that *was extremely soft*.	Ruth was a mother of three. Her first two children were in the early years of primary school and her third baby was now two months old. She was coping well with the additional responsibilities. But Ruth was so used to taking care of her older children that she almost forgot how different a baby's needs are. Ruth was on the look for new baby products and she heard about **bamp** – a new type of tissue that *was extremely soft*.
		2	Nick felt like he was ill every day of the year. He had a pretty bad immune system and needed to visit his doctor regularly for a whole range of problems. But fortunately, this didn't stop Nick from getting on with	Ruth continued doing the school runs for her children as her husband started work at 7am every day. In the beginning, Ruth struggled with the lack of sleep during the night, but she was now getting into a routine. On her

Item	Set	Paragraph	High CD	Low CD
			things. However, this time Nick felt extremely ill. He was suffering with a terrible headache and a cold with a runny nose. He knew it would be weeks before he would feel better. He liked to use a **bamp** for his nose because it was *scented with aloe vera*.	way back home from dropping the kids off, Ruth popped into her local supermarket and scanned the aisles for **bamp**. A customer told Ruth she really liked the product as it was *scented with aloe vera*. So, Ruth decided she would pick a packet up to try.
		3	Max was a plumber by trade. He started off installing and repairing parts for family and friends before he realised he was actually good at it. Max set up his own company and it was very successful. He received a call in the morning for an emergency kitchen repair and thankfully, the house was not too far from his own home. Once he got there, he set up his equipment and went to have a look. He saw that person who had called him used a **bamp** to soak up the leaking water. *He was surprised that it was very absorbent*.	Later that day, Ruth made herself a chicken Caesar salad for lunch while the baby was asleep. Ruth preferred eating salads on warm days rather than hot food. The summer seemed to be lasting longer than usual. Ruth heard a loud cry on the baby monitor, so she quickly went upstairs to check. Her baby had vomited all over his clothes! She'd only put that set on a few hours ago. Ruth used a **bamp** to clean the mess up. *She realised it was very absorbent because she only needed one*.
		4	Julie was watching television. Her favourite crime drama show was on and she sat there watching it with a mug of hot chocolate. Julie was into everything mystery and crime related and could binge watch entire series at once. When the advertisements came on, Julie popped to the kitchen to grab a biscuit and when she came back, she saw an advertisement for **bamp**. *They mentioned*	After the children had been picked up from school, Ruth began preparing some snacks for them to eat while they watched a cartoon show. Sam was fussy with most things in life and this included food. He only liked to eat plain bread and butter after school. Ruth gave the children their plates and after washing up, she used a **bamp** to wipe her hands. *She was going to tear it as she didn't*

Item	Set	Paragraph	High CD	Low CD
			that it was hard to tear, and Julie was intrigued.	*need an entire piece but noticed that it was hard to tear*.
		5	Alexa was in her last year of college. It was almost the end of year and she felt very stressed about revision. However, she did feel confident that her hard work up to this point would pay off. Alexa was in bed scrolling through Twitter on her phone. She liked to do this before bed because she could catch up with recent trends and laugh about the funny things people tweeted. She saw a post about **bamp** *and how it was made of panda poo*. She used them herself but now felt uncomfortable at the idea of even touching it.	By 8 o'clock, all the children were fast asleep. Ruth reflected on her day while she sat on the sofa with her husband. She thought it had been successful, but she was very exhausted and wanted to get into bed herself. She quickly tidied up the last bit of mess the children left at dinnertime and went upstairs. While Ruth brushed her teeth, she read the packet of the **bamp**. *She saw that it was made of panda poo*—probably not the best to directly use on her baby's sensitive skin.
Hoad	B	1	Austin and Kate had been married for five years. They'd recently bought a beautiful four-bedroom home in a prime location after saving for many years. Kate was especially interested in interior design and was really involved with decorating their home. But, they both felt the home was incomplete without a family pet. So the couple went to a pet store in town and were immediately attracted to a **hoad**. This was a type of <u>dog</u> that was *extremely furry*.	Austin and Kate had been married for five years. They'd recently bought a beautiful four-bedroom home in a prime location after saving for many years. Kate was especially interested in interior design and was really involved with decorating their home. But, they both felt the home was incomplete without a family pet. So, the couple went to a pet store in town and were immediately attracted to a **hoad**. This was a type of <u>dog</u> that was *extremely furry*.
		2	Martha had a busy few weeks coming up. It was the summer break and Martha and her family were going away for a two-week	The couple decided they would look around the store for a while before making their final decision. The store was large, and they

Item	Set	Paragraph	High CD	Low CD
			beach holiday. They had booked the holiday months in advance and were all really looking forward to it. Martha needed to buy some things before they left, including holiday clothes for her and the children. She also needed to give the family **hoad** to her parents while they were gone. He was getting quite old, *they generally had an 8-year lifespan*.	ended up spending many more hours there than initially planned. Austin spotted one of his friends, John, and jumped up behind him. He worked there part-time and they hadn't seen each other in months. Austin and Kate said to John that they were looking for a pet and were considering a **hoad**. *John said they generally had an 8-year lifespan*.
		3	Colin was a final-year veterinary student. The course was five years and it was demanding academically, physically, and emotionally, but he enjoyed it nevertheless. He hoped to open his very own vet clinic nearby one day. For his final project, Colin decided he wanted to research a rare breed of animal. He contacted a well-established veterinarian who offered Colin the opportunity to work with a **hoad**. *He discovered they were only bred in North America*.	Austin and Kate ended up leaving the store because they could not come to a final decision. They hoped to return soon. By the time they came home, Kate was really hungry and decided they would eat takeaway tonight. She didn't have enough time or even sufficient ingredients to make dinner. After ordering the food on her laptop, Kate decided to do some research about the **hoad**. *She discovered that they were only bred in North America*.
		4	Charles lived with his family in a small rural town. His main source of income was from agriculture, following in his father's footsteps. He was accustomed to a quiet and calm way of living so the busy nature of cities never interested him. Charles went for strolls along the local clifftops every day. Being such a small town, Charles knew almost everyone in the community and one	It was the next day and the sun was unusually bright and warm for an autumn's day. Both Austin and Kate were getting ready for work. Kate was a manager in a large department store which was really useful because she could get significant discounts for all the recent home décor shopping she was doing. Kate mentioned to her colleague that she and Austin were

Item	Set	Paragraph	High CD	Low CD
			day he saw his neighbour Paul walking his **hoad**. *He noticed how it had distinct small teeth*.	looking to buy a **hoad**. Her colleague had seen one before and *noticed how it had distinct small teeth*.
		5	Joanna was a fitness instructor. She worked in her local gym holding daily fitness classes and would even hold some classes free of charge because she wanted physical exercise to be accessible for all. Joanna was really passionate about her job. One day, Joanna woke up extra early in the morning, so she decided she would go for a run in the park behind her house with her **hoad**. A passer-by complimented its *bright green eyes* while Joanna stopped for a short break.	Austin and Kate decided to meet up after work and travel home together. Austin worked as a lawyer and was telling Kate that he felt really optimistic about an interesting case he was working on. He really loved his job and as he spoke, Kate couldn't help but notice they were passing the pet store in town. Kate didn't want any delay so they made their final decision and bought the **hoad** with no regrets. *They especially loved its bright green eyes*.
Coft	B	1	Nathan was doing his weekly grocery shopping in the supermarket. He liked to do this on Sunday so he had food for the rest of week. Nathan's flatmate was a really good cook and he would end up cooking for them both some nights in the week. The store was especially busy today as some tills were not working. Before Nathan left, he spotted a packet of **coft**. This was a new type of <u>cigarette</u> *that was made in Sweden* and he was keen to buy a packet.	Nathan was doing his weekly grocery shopping in the supermarket. He liked to do this on Sunday so he had food for the rest of week. Nathan's flatmate was a really good cook and he would end up cooking for them both some nights in the week. The store was especially busy today as some tills were not working. Before Nathan left, he spotted a packet of **coft**. This was a new type of <u>cigarette</u> *that was made in Sweden* and he was keen to buy a packet.
		2	Jacob was on his way back from an evening at his friend's house. Along with other friends, Jacob had spent the evening eating, drinking, and catching up. He liked to do	After Nathan got home, he set out his food purchases on the kitchen counter. He'd recently got into meal prepping, where he would plan meals for breakfast and lunch

Item	Set	Paragraph	High CD	Low CD
			this regularly to maintain a social life. He could have taken a bus or a taxi to get home but he wanted to go for a walk. It would take him around 30 minutes which wasn't too bad. He saw a lady on the pavement with a **coft** in her mouth. Interestingly, *the smoke contained blue pigments* and he was curious so he asked where it was from.	mainly. Nathan hadn't yet got into making food for every meal of the week because he had a busy schedule so sometimes he would eat out or on the go. He picked the **coft** packet out of the shopping bag and wanted to try one out. Nathan was really surprised to see that *the smoke contained blue pigments*.
		3	Zoe had worked as an administrative assistant for a financial services company for over a year. She knew she wasn't reaching her full potential in her current position, so she was applying for jobs internally in the organisation and outside the organisation. But she really liked the flexibility of her job. For example, if she came into the office early, she could also leave early. Zoe went out the building for regular breaks to smoke a **coft** *which didn't require a lighter—instead you had to peel and rub the tip to light it*.	Nathan enjoyed doing his meal prep because he found the process was quite therapeutic. He liked having things in order and being organised. Nathan's flatmate came back from the gym and asked what Nathan was making for this week. His main meal was grilled salmon with quinoa and broccoli. And for breakfast, he mainly chopped up different kinds of fruits. Nathan excitedly showed his flatmate his new **coft** *which didn't require a lighter—instead you had to peel and rub the tip to light it*.
		4	Adrian was feeling especially bored at home. His sister would usually play with him during the weekend, but she was at a sleepover at her friend's house. Adrian got off the sofa and decided he would look for things to do in the house. He liked to roleplay, so he went to the living room and gathered his toys. He pretended his toys were cowboys in the Wild West being	Nathan's flatmate would regularly remind him to stop smoking and Nathan knew it was important, but it would take time. He'd started smoking when his university friend introduced him to it and now it had been over 10 years. It was a very hard habit to break out of. The next week, Nathan tried to occupy his mind with other things such as going for runs in the early morning or the

Item	Set	Paragraph	High CD	Low CD
			confronted by the town sheriff. Adrian found a **coft** in his dad's coat pocket and put it in the sheriff's mouth. *It had a very unusually long shape*.	gym after work. But he found himself reaching for a **coft** frequently. *It had a very unusually long shape*.
		5	Casper was a builder in his late forties. Every weekday was the same for him. For his current building project, he would wake up and get ready at 6am, pick up a black coffee and get on the 6:45am bus to get to the building site for 7:30am. But Casper was recently suffering from chest problems. He told his doctor that he was a heavy smoker and he liked to smoke a **coft** *because you could reuse the same one up to five times*. His doctor suggested he should take some time off work to focus on his health.	Nathan's sister's wedding was coming up soon and he was very involved in the preparation. He was her only sibling, so they grew up as best friends and continued to be close. He couldn't believe she was getting married. Nathan joined his sister and her fiancé to try potential wedding cake flavours at the wedding venue. Her sister chose a well-established baker. Nathan stepped out of the room for a break and pulled out a **coft**. *He thought it was useful that you could reuse the same one up to five times*.
Veak	B	1	Claire was a secondary school student. She was in her last year and would soon be leaving for college. Claire really enjoyed her time at school. She knew she would miss her school friends, her home town, and even her classes. She particularly enjoyed science and was fascinated by understanding how things work the way they do. Her latest project for her biology class was to examine a **veak**. This was a type of <u>cockroach</u> *that only lives in New Zealand*.	Claire was a secondary school student. She was in her last year and would soon be leaving for college. Claire really enjoyed her time at school. She knew she would miss her school friends, her home town and even her classes. She particularly enjoyed science and was fascinated by understanding how things work the way they do. Her latest project for her biology class was to examine a **veak**. This was a type of <u>cockroach</u> *that only lives in New Zealand*.
		2	Jonathan was spending the evening indoors. His day at work had been busy so he wanted	Claire was heavily involved in extra-curricular activities at school. She was part

Item	Set	Paragraph	High CD	Low CD
			to relax and catch an early night's sleep. He had just placed an order for a cheese pizza and was planning to watch some television. He'd recently started watching a new nature programme. This was a 10-part series with a famous presenter travelling the world to discover rare species. The episode being aired that day was about **veak**. *The presenter mentioned they were able to fly*.	of the girls' basketball team as the head captain. Her team participated in competitions with other local schools and had won two awards in the past year. The next game was in a few days, but before spending time training for the competition, she focused on completing some of her work including her biology project. She kept the **veak** in a big glass jar because it *was able to fly*.
		3	Mike was eleven years old and was spending his summer holidays at his grandmother's house. The weather had been great with clear blue skies for the past few weeks, so Mike liked to spend most of his day outdoors. He made friends with the neighbourhood children and they would usually go to the park behind their street to play football till sunset. One day, Mike accidently crushed a **veak** with his foot. He went closer to inspect it and *discovered that it releases a very bad smell*.	One day at school, Claire sat with her friends in the lunch canteen. She had known her best friend since she was three years old and did everything with her. Claire was not very fond of the food generally served at her school, so she just opted for a simple cheese and tomato sandwich that day. After she finished the sandwich, Claire remembered that she needed to quickly check on the **veak**. She opened the lid of the jar and *discovered that it releases a very bad smell*.
		4	Louise was a scientist who worked in a laboratory specialised in genetic research. She had been working as a research assistant through an internship after university. Louise's manager really admired her work ethic and intelligence so offered her a permanent position in a more senior role. She had been working there for five years	On her way back to her class for registration, Claire saw her biology teacher, Mr Jones. He was her favourite teacher, especially because of his interactive style of teaching. Mr Jones had been her teacher for the past five years, so he knew Claire very well. He had written her reference for the college she was accepted into. Claire

Item	Set	Paragraph	High CD	Low CD
			and really enjoyed her job. Louise and her colleague's current project focused on the genetics of the **veak**. She knew that it *had a rapid life cycle*.	mentioned that her research about the **veak** was going well. She'd found out that *it had a rapid life cycle*.
		5	Seth was having a terrible weekend. On Saturday morning, he woke up ill with a sore throat and runny nose. Later that day, Seth walked into his garden to find that all his plant pots had been knocked over. It was probably his next-door neighbour's mischievous cat! And then on Sunday, Seth's son woke up him up to remove a **veak** he found. He needed to be careful because *they carry disease*. Unfortunately, Seth discovered his home was in fact infested and he would need an exterminator.	Before the end of the day, Claire needed to hand in an essay to her history teacher. They were working on the Cold War and the role of the Soviet Union. Claire hoped she would get a good mark on this essay as she was rather disappointed with her previous result. Claire also needed to head back to the science classroom to take some pictures for a presentation she would be doing on her research. *But she remembered to be careful when handling the* **veak** *because they carry disease*, so she used rubber gloves.
Hust	B	1	Matt was a consultant at a large city firm. It was Friday and his manager asked if he was available to attend a conference over the weekend as Matt's colleague had dropped out last minute. Matt didn't mind very much because he didn't have plans for the weekend and he would be paid for his time. But he had only the evening to quickly pack a small hand luggage. To utilise the limited space, Matt packed a **hust** – a new brand of shirt *that came in many different print patterns*.	Matt was a consultant at a large city firm. It was Friday and his manager asked if he was available to attend a conference over the weekend as Matt's colleague had dropped out last minute. Matt didn't mind very much because he didn't have plans for the weekend and he would be paid for his time. But he had only the evening to quickly pack a small hand luggage. To utilise the limited space, Matt packed a **hust** – a new brand of shirt *that came in many different print patterns*.

Item	Set	Paragraph	High CD	Low CD
		2	Jane had just finished college and recently qualified to be an air hostess. Her first flight was to Australia, a place she'd always wanted to visit. She would be there for three days so there would be plenty of time to explore Sydney. Jane was fitted for her new uniform which included a **hust**. She knew this would save her plenty of time when getting ready as *it was non-iron with virtually no creases*.	There was a lot of traffic on his way in because of a road diversion, so Matt got to his hotel at 1am. Thankfully, checking in was smooth as Matt's manager had swapped over the details. However, the late night meant he woke up late the next morning and had to quickly get ready to arrive at the conference venue on time. The **hust** *he brought with him was non-iron with virtually no creases*. He was glad about this because there were no irons available in the hotel and he didn't have any time to iron anyway.
		3	Being a first-time mum, Clara didn't have much time to declutter the house to make space for new things for the baby. But Clara was finally looking forward to doing some spring cleaning. She started off by clearing out her wardrobe because many of her clothes were worn out. As it happened, her friend Susan came over for a cup of tea and suggested she should buy a **hust** *which was stain-proof*. Clara thought this was perfect for her because it would mean she wouldn't always be covered in baby mess.	The morning of the conference went very well. Matt learned a lot about the new client and the areas they needed consulting services in. He felt confident that his team could apply their knowledge and experience to help. Matt made notes to take to his manager when he returned to the office on Monday. He left his fountain pen in the pocket of his **hust** but the ink leaked! He didn't worry as *it is stain-proof*, so the ink would easily come out.
		4	Mr Stanley was a teacher in a secondary school in London. This was the third school he'd worked at and had just been made Head of Department. He was slowly getting used to the increased responsibility. At the school, David was known for his eccentric style of	At lunch time, all the conference guests were taken to the open buffet and the food selection was very good. Matt spent the time networking with his peers and ate lots of food, both sweet and savoury. However, he remembered he wanted to be mindful about

Item	Set	Paragraph	High CD	Low CD
			dressing among students and staff alike. At lunch time, his colleague Mr Guzman, a Spanish teacher, complimented his **hust**. David explained it was very comfortable because *the material is stretchy*.	his calorie intake because he was worried about putting on weight. Some of his clothes were not fitting him well anymore, which is why he preferred wearing his **hust** as *the material is stretchy*.
		5	Yuki was a Japanese fashion designer who had been working in the industry for 15 years. Fashion was something he'd always had a passion for and it was his dream to become one of the top designers of innovative clothing. His brand had been highly successful with some of his pieces even appearing in London Fashion Week. His line focused on practicality and one of his pieces included a **hust** *which had a tear proof design*.	Finally, the conference was over, and he wanted to get home as soon as possible to make use of the rest of the weekend. But Matt remembered the road diversion would extend his journey time. He would definitely need keep alert, so he stopped at the nearest service station and bought a cup of coffee and a few snacks. When returning to his car, Matt dropped his wallet and as he bent down to retrieve it, his **hust** got caught on the corner of his car door. *Luckily, it was tear proof so there was no damage*.
Deam	B	1	The winter holidays had officially started. Tim was especially looking forward to this holiday because his dad booked a cabin for the family. He hoped to play in the snow all day. Once they arrived at the cabin, the children ran to choose their rooms. Tim didn't mind what room he would be sleeping in—instead he looked around the place. Outside, he found an unlocked shed full of all sorts of things. As he stepped out, he spotted a **deam** which was a type of <u>sled</u> and *its design was based a snow board*.	The winter holidays had officially started. Tim was especially looking forward to this holiday because his dad booked a cabin for the family. He hoped to play in the snow all day. Once they arrived at the cabin, the children ran to choose their rooms. Tim didn't mind what room he would be sleeping in—instead he looked around the place. Outside, he found an unlocked shed full of all sorts of things. As he stepped out, he spotted a **deam** which was a type of <u>sled</u> and *its design was based a snow board*.

Item	Set	Paragraph	High CD	Low CD
		2	Steven was thirty years old. He lived by himself in a small apartment in town and he was a minimalistic man who had everything he needed. Steven met his friends regularly for nights out such as for drinks or to go to the cinema. Steven worked as a full-time designer in a manufacturing company which made all sorts of things. The most recent product he and his team were working on was a **deam**. *It was designed to be large enough for one adult or two children*.	The owners of the cabin decorated it very nicely for the festive period. In the main living space, the walls were lined with garlands and there was a large Christmas tree with numerous ornaments. Before bedtime, the family sat in front of the wooden fireplace to get warm. Tim thought the place was very welcoming and he thought about how he couldn't wait to play with the **deam** in the morning. *It was large enough for one adult or two children*, so maybe his brother could join him.
		3	Stella recently found a job in a retail store nearby. It was only a short drive away and she needed as a job as soon as possible. She was saving for a girls' trip with her friends and needed money quickly. Stella's new manager was nice, and she was given decent working hours. She was tasked with bringing in deliveries during her morning shift. She didn't really like doing this task because many of the products that came in were heavy. But today, Stella brought in **deam** packages and she easily managed because *they are very lightweight*.	Tim woke up bright and early and was ready for the day to begin. He gobbled down toast with scrambled eggs and a glass of orange juice for breakfast. His mum told him to slow down but he just couldn't wait to go outside and play. He put on his best snow boots and ran outside. He sighed when his mum immediately called him back in… he forgot his gloves. Tim walked up a small hill outside the cabin with the **deam**. He easily managed because *it was very lightweight*.
		4	Janet was eight years old and she was a fun and confident girl. During the weekends, Janet went to dance class—she was very good at contemporary dance and ballet. And after school, Janet liked to take part in	It had been hours since Tim began playing outside, and his siblings had joined him. Tim got along really well with his family. He was particularly close to his brother because they had very similar interests and hobbies. Tim

Item	Set	Paragraph	High CD	Low CD
			sporting activities, such as netball. It was the end of the first term and Janet's primary school organised a sports day. Some activities were indoors while other activities were outdoors. Janet ended up winning the **deam** racing for her class and she thought *the steering wheel for manoeuvring was very helpful*.	and his brother only had a one-year age gap while the age gap with his sister was four years. The siblings competed to see who could get down the hill the fastest on the **deam**. *Tim thought the steering wheel for manoeuvring was very helpful*, and he ended up winning three rounds out of five.
		5	It was almost Christmas day and Josh hadn't yet bought all the gifts he needed to buy for his family and friends. It wasn't that they were difficult to buy for but Josh just didn't have the time. On Saturday morning, he decided it was time he went to the shops and bought everything while the rest of the family were asleep. His son, Todd, requested a **deam** for his present. When Josh got to the store, he saw that it was *surprisingly cheap because they are mass produced*.	The rest of the holiday seemed to fly by and it was the family's last day at the cabin. Tim was especially sad to leave as he'd had such a great time playing and having fun. Winter was his favourite time of year because he loved layering up and spending time outside but also being cosy indoors watching films. He wasn't ready to go back to school. To cheer Tim up, his dad promised that he would get him his very own **deam**. His dad saw that it was *surprisingly cheap because they are mass produced*.
Zove	B	1	Olivia, Abigail and Mia had been best friends ever since high school. They did almost everything together and were inseparable. Even after finishing high school, the girls kept in touch and met regularly during university. It had been five years since all of them had graduated, and they were finally going on the road trip across the country that they had planned many years ago. The three of them decided	Olivia, Abigail and Mia had been best friends ever since high school. They did almost everything together and were inseparable. Even after finishing high school, the girls kept in touch and met regularly during university. It had been five years since all of them had graduated, and they were finally going on the road trip across the country that they had planned many years ago. The three of them decided

Item	Set	Paragraph	High CD	Low CD
			they would take turns driving Olivia's new **zove** on the journey. It was a new type of car that *only fit three people*, which was ideal for them of course.	they would take turns driving Olivia's new **zove** on the journey. It was a new type of car that *only fit three people*, which was ideal for them of course.
		2	It was Alex's first week of school. His mum, Amy, was feeling very stressed because he didn't seem to be settling in well. He cried every morning when she dropped him off and his teacher said he was very shy and quiet in class. Alex would only be going in for half the day at the start of the term and the teachers reassured Amy that many children are like this and soon become comfortable. At home time on Friday, Amy drove her **zove** to pick up Alex from school. Unfortunately, Alex spilled his chocolate milk all over the *suede seats*.	It was the day the girls' road trip would start and Olivia woke up early in the morning. She could hardly contain her excitement. She was really looking forward to relaxing, spending time with her friends, and not having to worry about work. When she was with Abigail and Mia, it felt like they were high school students again. Mia stayed the night at Abigail's house, so Olivia went to pick them up. They put all their belongings in the boot of the **zove** and realised they'd definitely overpacked! Abigail complimented the *suede seats* as she stepped in.
		3	Jonathan lived far from his parents. He decided to move to the city to study and ended up living there permanently because he had secured a very good job. He visited his parents as often as he could which was at least once a month. The route to Jonathan's parents' home wasn't the safest as some the roads were mountainous without barriers. One day he was stranded because his **zove** broke down. But luckily, *it had a built-in hot beverage maker*, so he could keep warm with some coffee until help arrived.	It was four hours into the journey and the girls were having a blast. Abigail was in charge of the music and she was playing old school throwbacks. Olivia couldn't help but laugh at their attempts to sing. It would soon become night and Olivia wanted to maintain her concentration while driving. Mia offered to take over the driving, but Olivia decided she could continue a little further for now. Olivia's **zove** *had a built-in hot beverage maker*, so she asked Mia to make her a coffee to help her stay alert.

Item	Set	Paragraph	High CD	Low CD
		4	Leo was always late for work. He felt like no matter how hard he tried, the clock was always against him as one thing or another would go wrong. Today he woke up with ample time to get dressed, eat a plentiful breakfast, and be on his way. However, there was so much traffic on the motorway due to a collision ahead that Leo got stuck in standstill traffic in his **zove**. He decided to *remove the steering wheel to enable auto-drive*. There was nothing else he could do to get to work faster.	Olivia had been carefully planning their route months in advance, so she knew where they would be spending the nights. They were headed for a motel which wasn't very far. Mia searched the motel name on her phone and saw people had given it decent reviews. She hoped it would be clean. Olivia's **zove** was driving at a slow speed due to the traffic, so she *removed the steering wheel to enable auto-drive*. She could give her arms a rest at least.
		5	James worked for a relatively new company. He had many sleepless nights and worked tirelessly because he really wanted to see the company succeed and develop. He felt like everyone's effort was finally paying off because they were making great sales and receiving amazing recognition. His team were specifically involved in engineering the **zove** in innovative ways. One of its most interesting characteristics was that *it was partially wind-powered and sustainable*. The company won a major energy award for this.	The girls finally arrived at the motel at 9 o'clock in the evening. The motel was pretty busy, but that was expected as it was summer. Luckily, they managed to secure the last triple bedroom left. The girls were really hungry, especially Abigail, despite her eating the most snacks throughout the journey. The owner of the motel told her that there was a local diner only a two-minute drive away, so they decided to head there in Olivia's **zove**. She mentioned that *it was partially wind-powered and sustainable*.
Yark	B	1	Hailey was finally free. She'd just stepped out of the main hall in school where her last GCSE exam was being held. It had been a very tough month for her. Hailey was revising constantly, so her family hardly saw her. She hoped her hard work would pay off	Hailey was finally free. She'd just stepped out of the main hall in school where her last GCSE exam was being held. It had been a very tough month for her. Hailey was revising constantly, so her family hardly saw her. She hoped her hard work would pay off

Item	Set	Paragraph	High CD	Low CD
			but now it was time to celebrate. Hailey and her friends organised a picnic for later that afternoon. Hailey was going to bring her **yark** which was a type of <u>frisbee</u> *that could withstand strong winds because it had the shape of a hoop*.	but now it was time to celebrate. Hailey and her friends organised a picnic for later that afternoon. Hailey was going to bring her **yark** which was a type of <u>frisbee</u> *that could withstand strong winds because it had the shape of a hoop*.
		2	Jaden was 7 years old. He loved watching television, playing videogames, and eating junk food. But he liked to spend most of his time outdoors with his best friend who was also his next-door neighbour. He was more like a brother to Jaden. It was Jaden's birthday party last week and his family, friends, and relatives bought him a whole load of presents. The next Monday was 'show and tell' in school and Jaden wanted to show one of his presents. He decided to bring in his new **yark** *which came in 30 different colours*—his one was red.	Hailey and eight of her friends were going to the picnic but first they all headed home to get out of their school uniform. Hailey's mum asked how the exam went. Hailey thought she could have revised certain topics more, but she didn't want to dwell on it. She put on a simple floral dress with a denim jacket and white trainers. Hailey left the house and had been walking towards the park for two minutes before she had to turn around because she forgot the **yark**! *It could be bought in 30 different colours*, and her one was red.
		3	It was 7pm and Owen was preparing dinner. The clocks went back an hour so it was getting dark earlier. Owen's favourite season was winter because he thought it was cosier and he liked staying indoors. Owen was making spaghetti bolognese with cheesy garlic bread. Suddenly, the electricity in his house cut off and everything became pitch black. Owen's phone was running out of battery so he couldn't use the torch, but he	Hailey was on her way again and she stopped at a local shop because she was tasked with bringing the drinks for the picnic. She put a few bottles of soft drinks, juices, and water in her basket and went to pay for them. Once she got to the park, she realised she was the first one there, so she sat on the grass waiting for the others. She also forgot to bring a picnic blanket—hopefully the others would bring enough! She took the **yark** out of her bag and

Item	Set	Paragraph	High CD	Low CD
			used his **yark** instead. *It always glowed, in both the day and night*.	examined it. *It always glowed, in both the day and night*.
		4	It was Saturday morning and Ryan was playing in his front yard after breakfast. The front yard was fairly large so Ryan could kick his ball around. Ryan saw his friend Thomas walking towards him on the street. Thomas moved here from another town three years ago and Ryan immediately became his friend as they got along really well. Thomas told Ryan he could only play with him for a short while as he had to head off to visit his aunt soon. Ryan wanted to show Thomas his new **yark** so he went indoors to retrieve it. He explained that *it was made of unbreakable material*.	Hailey's friends finally arrived, and they set up all the food for the picnic such as sandwiches, crisps, chocolate. They were all so happy that the year was finally over, and they could just relax for the next few months. Hailey told her friends how excited she was that her family were going away to Spain in August. When they finished eating, the friends started playing cards. One of her friends was really happy about winning the round so she jumped up in joy. She accidently stood on the **yark** but Hailey said not to worry as *it was made of unbreakable material*.
		5	Melanie was going to her grandparents' house with her parents for the weekend. She was really close to her grandparents because they'd lived with them for a few years when she was younger. Melanie loved her grandparents' house because it was so large and there was so much more to do in the area. She'd packed enough clothes for the weekend and she'd also packed her **yark** *which was light as a feather*. She thought she could play it with her grandparents' dog.	Hailey's friend Jocelyn suggested they should all race from one side of the park to the other. Hailey came second place and she was really out of breath. She'd hardly exercised these past few weeks. Normally she liked to go for runs in her area and even joined the local gym a few months ago. She thought that it was time for her to re-join the gym. Hailey brought out her **yark** and everyone started playing with it. Her friend was surprised to find that *it was as light as a feather*.
